# T and B cell abnormalities, pneumocystis pneumonia, and chronic lymphocytic leukemia associated with an AIOLOS defect in patients

**DOI:** 10.1084/jem.20211118

**Published:** 2021-10-25

**Authors:** Hye Sun Kuehn, Jingjie Chang, Motoi Yamashita, Julie E. Niemela, Chengcheng Zou, Kazuki Okuyama, Junji Harada, Jennifer L. Stoddard, Cristiane J. Nunes-Santos, Brigette Boast, Ryan M. Baxter, Elena W.Y. Hsieh, Mary Garofalo, Thomas A. Fleisher, Tomohiro Morio, Ichiro Taniuchi, Cullen M. Dutmer, Sergio D. Rosenzweig

**Affiliations:** 1 Immunology Service, Department of Laboratory Medicine, Clinical Center, National Institutes of Health, Bethesda, MD; 2 Laboratory for Transcriptional Regulation, RIKEN Center for Integrative Medical Sciences, Kanagawa, Japan; 3 Department of Pediatrics and Developmental Biology, Graduate School of Medical and Dental Sciences, Tokyo Medical and Dental University, Tokyo, Japan; 4 Department of Immunology and Microbiology, University of Colorado School of Medicine, Aurora, CO; 5 Division of Allergy and Immunology, Department of Pediatrics, University of Colorado School of Medicine, Children’s Hospital Colorado, Aurora, CO

## Abstract

AIOLOS/*IKZF3* is a member of the IKAROS family of transcription factors. IKAROS/*IKZF1* mutations have been previously associated with different forms of primary immunodeficiency. Here we describe a novel combined immunodeficiency due to an *IKZF3* mutation in a family presenting with T and B cell involvement, *Pneumocystis jirovecii* pneumonia, and/or chronic lymphocytic leukemia. Patients carrying the AIOLOS p.N160S heterozygous variant displayed impaired humoral responses, abnormal B cell development (high percentage of CD21^low^ B cells and negative CD23 expression), and abrogated CD40 responses. Naive T cells were increased, T cell differentiation was abnormal, and CD40L expression was dysregulated. In vitro studies demonstrated that the mutant protein failed DNA binding and pericentromeric targeting. The mutant was fully penetrant and had a dominant-negative effect over WT AIOLOS but not WT IKAROS. The human immunophenotype was recapitulated in a murine model carrying the corresponding human mutation. As demonstrated here, AIOLOS plays a key role in T and B cell development in humans, and the particular gene variant described is strongly associated with immunodeficiency and likely malignancy.

## Teaser

We report four patients in a multigenerational family carrying a novel heterozygous variant in AIOLOS/IKZF3 associated with T and B cell developmental and functional defects, CID, PJP, and CLL. Identification of germline IKZF3 mutations introduces us to hereditary AIOLOS-associated diseases in humans.

## Graphical Abstract

**Figure:**
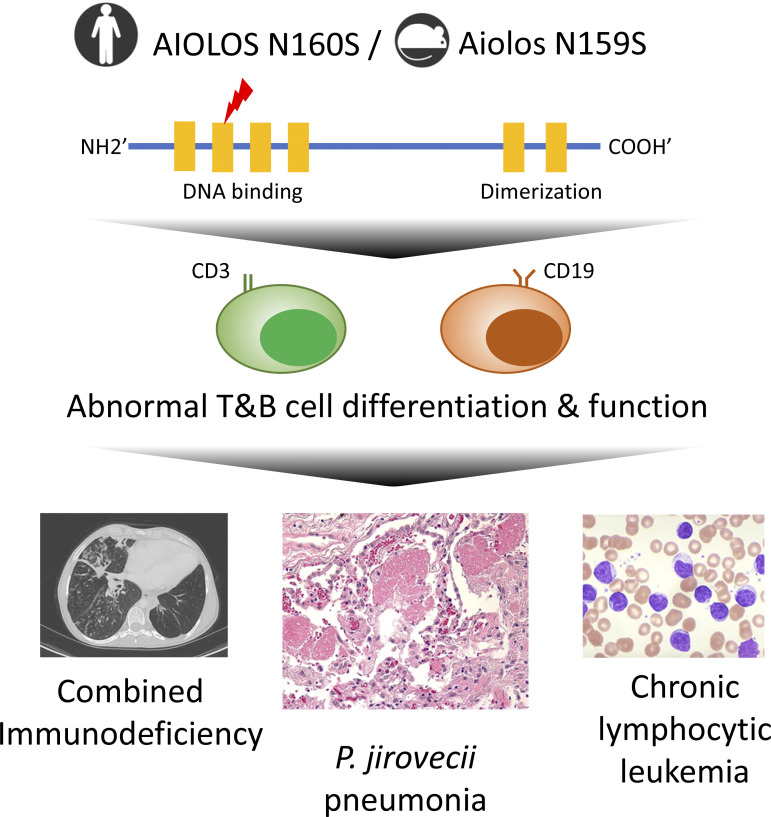

## Introduction

Aiolos, an Ikaros family transcription factor encoded by *Ikzf3*, plays a pivotal role in murine B cell differentiation (Morgan et al., 1997; Wang et al., 1998). Both Aiolos and Ikaros have been shown to be important regulators of lymphocyte development and differentiation (Georgopoulos et al., 1997; Wang et al., 1998; Wang et al., 1996). While Ikaros expression is detected in myeloid and erythroid precursors as well as in pluripotent hemopoietic stem cells, Aiolos is first detected in early lymphocyte precursors, but not in myeloid and erythroid precursors (Georgopoulos et al., 1994; Morgan et al., 1997). The expression levels of Aiolos are low in pro-B cells and double-negative thymocyte precursors but are greatly up-regulated in pre-B cells and double-positive T cells (Morgan et al., 1997; Wang et al., 1998). Abnormal B cell differentiation and functions were observed in Aiolos-deficient mice, including increased numbers of B cell precursors, breakdown in B cell tolerance, spontaneous autoantibody production, and the development of B cell malignancies, suggesting the important role of Aiolos in B cell differentiation and development (Wang et al., 1998). Like Ikaros, full-length Aiolos contains four N-terminal zinc fingers (ZFs) that mediate DNA binding and two C-terminal ZFs that facilitate homo- and heterodimerization with Ikaros family members (Caballero et al., 2007; Morgan et al., 1997). Aiolos is also known to affect transcriptional regulation through chromatin remodeling by homodimerization or heterodimerization with Ikaros or Ikaros family members (Kim et al., 1999; Morgan et al., 1997).

In the past few years, several studies have shown that germline *IKZF1* mutations are associated with immunodeficiency. Depending on the allelic variants and the mechanisms of action, the resulting clinical and immunological phenotypes vary. The majority of patients with IKAROS haploinsufficiency mutations present with B cell deficiency, hypogammaglobulinemia, increased susceptibility to bacterial infection, and autoimmunity/immune dysregulation, a phenotype compatible with common variable immunodeficiency (CVID). In contrast, patients with dominant-negative variants manifest with a more aggressive combined immunodeficiency (CID) phenotype including B and T cell developmental defects and increased susceptibility to recurrent and opportunistic infections, particularly *Pneumocystis jirovecii* pneumonia (PJP). Finally, patients with dimerization defective mutations, which also act by haploinsufficiency, demonstrate a milder impact on B cell numbers and bacterial infection, but have a higher incidence of immune dysregulation/autoimmune diseases and hematologic malignancy compared with the other allelic variants (Kuehn et al., 2016, 2021a, 2021b; Boutboul et al., 2018).

Very recently, Yamashita et al. (2021) showed that the AIOLOS*/IKZF3* G159R mutation is associated with B cell deficiency and recurrent sinopulmonary infections, as well as EBV infection susceptibility and B cell lymphoma, through a dominant-negative effect over both AIOLOS and IKAROS WT protein function.

The patients in the family described here presented in childhood with symptoms of immunodeficiency suggestive of CID. We performed whole-exome sequencing (WES) and identified a novel heterozygous AIOLOS/*IKZF3* N160S mutation. Patient data and a mouse model with the corresponding mutation demonstrated that this AIOLOS (human)/Aiolos (mouse) mutation affects both T and B cell differentiation and function and, when presenting as a human disease, leads to an increased susceptibility to infectious diseases, mainly PJP and other sinopulmonary infections, as well as a likely increased risk for chronic lymphocytic leukemia (CLL).

## Results

### Clinical histories

The family studied here had four individuals in three generations affected with immunodeficiency, PJP, and/or CLL. Index patient A.III.1 was born at term and, during her first year of life, developed failure to thrive and respiratory infections, including PJP. Her initial immune evaluation detected severe hypogammaglobulinemia of all isotypes with a normal absolute B cell count. Her father (A.II.2), paternal aunt (A.II.1), and paternal grandfather (A.I.1) already carried the diagnosis of primary immunodeficiency since childhood. The father presented with recurrent respiratory infections during infancy followed by the identification of severe hypogammaglobulinemia at the age of 1 yr, after which he began IgG replacement and prophylactic antibiotics. The paternal aunt presented at 11 mo of age with bacterial pneumonia and meningitis, at which time she was found to have severe hypogammaglobulinemia; she has since received IgG replacement, and her subsequent clinical course has included recurrent sinopulmonary infections, oligoarticular arthritis, PJP (one episode), CLL (diagnosed at 30 yr of age), and metastatic melanoma (diagnosed at 35 yr of age). The paternal grandfather suffered from recurrent sinopulmonary infections since infancy and was found to have severe hypogammaglobulinemia at 14 yr of age (first assessment), at which time IgG replacement was initiated, and his subsequent clinical course has included PJP (one episode), extensive cutaneous warts, bronchiectasis, *Mycobacterium avium* complex lung disease, sepsis, and a skewed kappa/lambda ratio (8:1) in the peripheral B cell population. No EBV-associated diseases were reported in any affected family members.

### Identification of an *IKZF3* mutation and its functional testing

**Figure 1.**
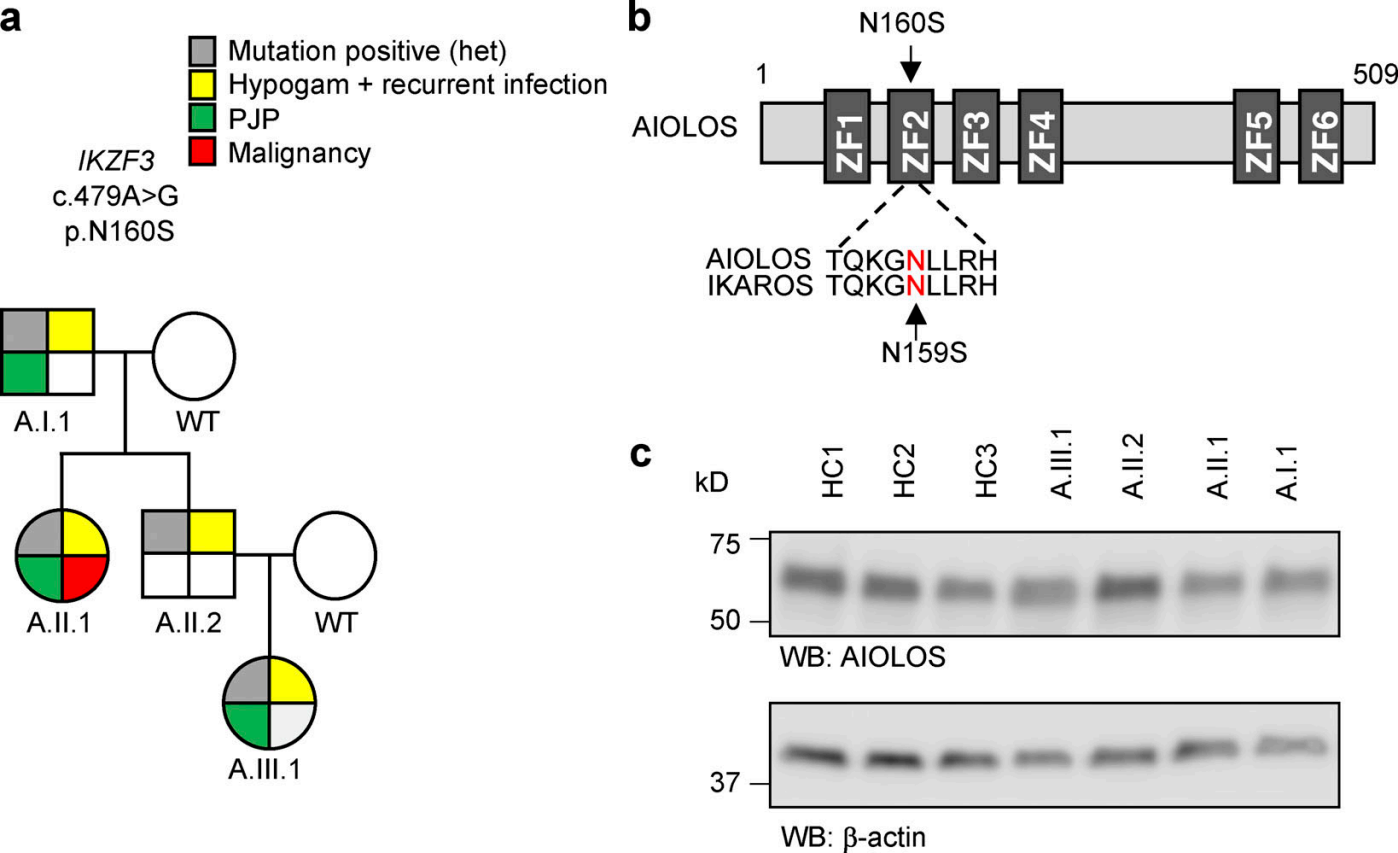
**Genetic information and pedigree of a family with the *IKZF3* mutation.** **(a) **Segregation of the *IKZF3* mutation and clinical and immunological phenotypes. Squares and circles indicate male and female family members, respectively. Hypogam, hypogammaglobulinemia. **(b) **Schematic presentation of the structure of AIOLOS isoform 1 (NCBI accession no. NM_012481). Dark gray indicates the ZFs (ZF1–6), and an arrow indicates the site of mutation. Numbers indicate the amino acid location. The previously reported germline IKAROS mutation (N159S) in patients with CID is indicated below the AIOLOS sequence. **(c)** AIOLOS protein expression levels were tested in T cell blasts from the patients with the AIOLOS mutation and healthy controls (HC). β-Actin was used as a loading control. WB, Western blot. Representative images from two independent experiments are shown.

We performed WES on samples obtained from the index patient and her parents and identified a germline heterozygous *IKZF3* mutation (NM_012481:c.479 A>G, p.N160S), located in ZF2 of the DNA binding domain, in the index patient and her father; variants were confirmed by Sanger sequencing (Fig. 1 a and Table S1). The AIOLOS N160S mutation stood out as the best candidate variant, because multiple sequence alignments of IKAROS and AIOLOS protein sequences implied that AIOLOS N160 is homologous to IKAROS N159, the site of an IKAROS variant (N159S) that previously has been associated with CID and PJP (Fig. 1 b; Boutboul et al., 2018). *IKZF3* is predicted to be under moderate purifying selection (neutrality index = 0.021). Moreover, two loss-of-function (LOF) *IKZF3* single-nucleotide variants are reported in gnomAD, with low LOF observed/expected upper bound fraction (0.2) and high probability of LOF intolerance (0.98). Taken together, these data indicate that *IKZF3* is a highly constrained gene that is extremely LOF intolerant and likely to be in the haploinsufficient (dominant) gene class (Table S1). Segregation analysis and Sanger sequencing confirmation were performed in all available family members, with the same AIOLOS N160S mutation detected in the paternal grandfather (A.I.I) and the aunt (A.II.1). We further performed WES on A.I.1 and A.II.1, and only one other rare immune-related gene variant in *JAK3* (c.2518C>T, p.R840C) was identified in all four affected family members, but not in the index patient’s mother. This *JAK3* heterozygous missense variant was present in 59 presumably healthy individuals in gnomAD and affects a gene for which functional or clinical haploinsufficiency has not been described (O’Shea et al., 2004). When JAK3-dependent STAT5 and STAT6 phosphorylation upon IL-7 (p-STAT5) and IL-4 (p-STAT6) stimulation was evaluated in three affected family members (A.II.1, A.II.2, and A.III.1), the results were comparable to healthy controls, therefore ruling out any major biological role for this JAK3 change (data not shown).

While evaluating the impact of the *IKZF3* c.479 A>G, p.N160S mutation, we first tested AIOLOS protein expression in T cell blasts that were generated by TCR stimulation. The protein expression level was comparable between healthy controls and all family members carrying this variant (Fig. 1 c).

**Figure 2.**
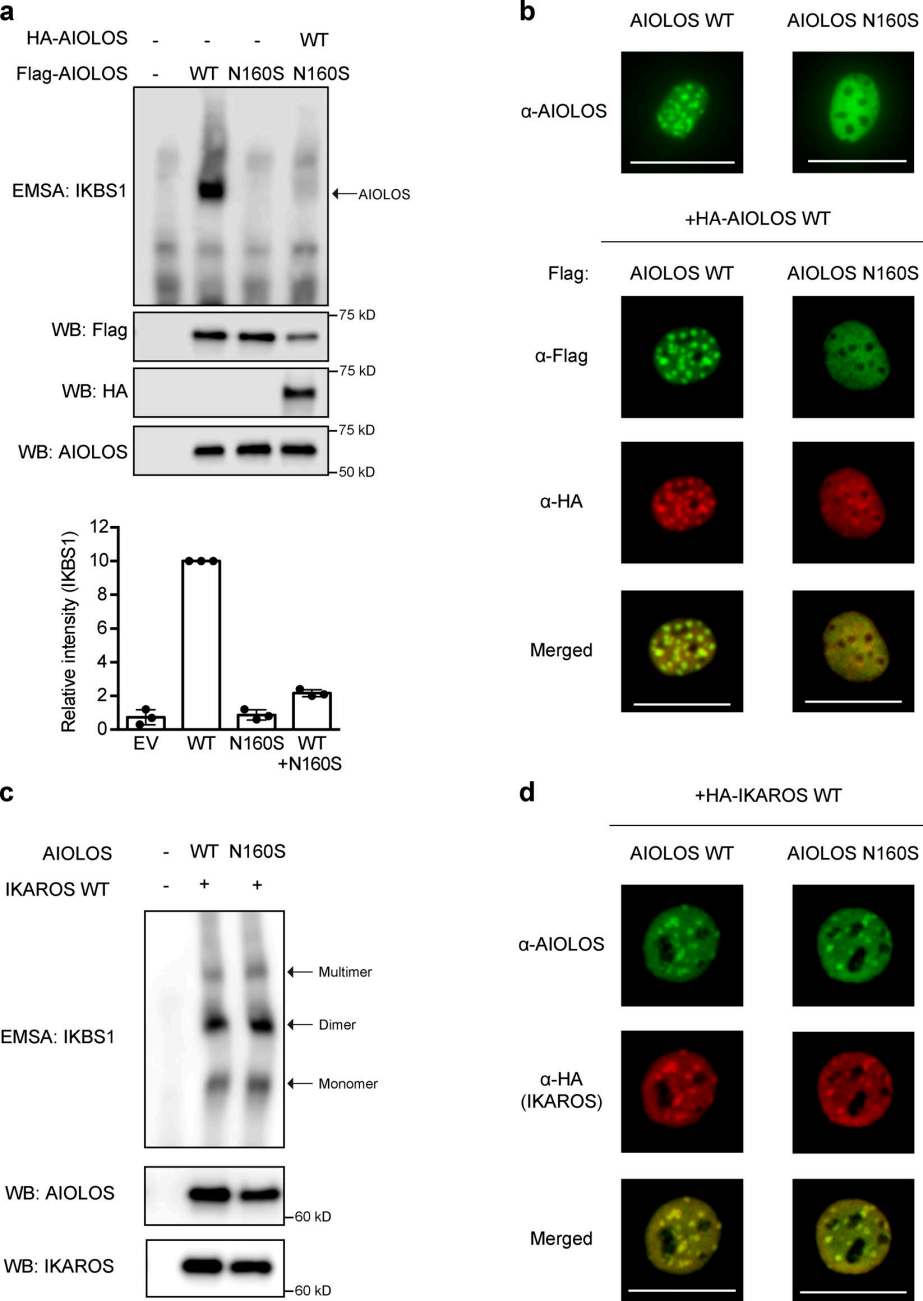
**Functional assessments for the AIOLOS mutation.** **(a) **EMSA was performed using nuclear extracts from HEK293T cells transfected with Flag-tagged AIOLOS WT and mutation, alone or together with HA-tagged WT AIOLOS as indicated in the figure. The AIOLOS WT and/or mutant expression levels from the nuclear extracts were similar between samples. The nuclear extracts were used for EMSA. Densitometry was performed to quantify AIOLOS binding to the IKBS1 probe, and values were normalized to the WT sample results. Data represent the mean ± SD of three independent experiments. WB, Western blot. **(b) **NIH3T3 cells were transfected with Flag-tagged WT or mutant expression vector, alone or together with HA-tagged WT AIOLOS. Cells were labeled with indicated antibodies followed by Alexa Fluor 488–conjugated (green) and/or Alexa Fluor 568–conjugated (red) secondary antibodies. **(c) **HEK293T cells were cotransfected with Flag-tagged AIOLOS WT or mutation together with Flag-tagged WT IKAROS. IKAROS and AIOLOS expression from nuclear extracts were tested using anti-IKAROS and anti-AIOLOS antibodies. The nuclear extracts were used for EMSA. Arrows indicate IKAROS-specific multiple binding sites. **(d)** NIH3T3 cells were transfected with HA-tagged WT IKAROS, alone or together with Flag-tagged AIOLOS WT or mutant expression vectors. Cells were visualized using an EVOS (20× objective) or ZOE (original magnification 175×) fluorescent cell imager. The scale bars indicate 25 µm. Data in panels a–d are representative of three independent experiments.

Next, vectors expressing WT AIOLOS and mutant AIOLOS N160S were tested to evaluate the functional impact of this variant. The ability of the mutant to bind its DNA target sequence was determined by the electrophoresis mobility shift assay (EMSA), demonstrating that WT AIOLOS bound normally to IKBS1 (a binding site shared with IKAROS) while mutant AIOLOS N160S failed to bind (Fig. 2 a). This finding is similar to the previously reported IKAROS N159S dominant-negative mutation (Boutboul et al., 2018). To determine whether the mutant AIOLOS N160S had a dominant-negative effect, WT AIOLOS and AIOLOS N160S were coexpressed in HEK293T cells. DNA binding was reduced by ∼80% in cells coexpressing AIOLOS WT/N160S compared with WT-only transfected cells (Fig. 2 a). Next, pericentromeric-heterochromatin (PC-HC) localization was tested to evaluate if there was a dominant-negative effect on pericentromeric targeting. Whereas the WT protein-expressing cells exhibited the expected punctate staining pattern indicative of PC-HC localization, the mutant failed to target to the PC-HC when expressed alone as well as when coexpressed with WT AIOLOS, suggesting that the mutation has a dominant-negative effect on both WT AIOLOS DNA binding and pericentric localization (Fig. 2 b).

**Figure 3.**
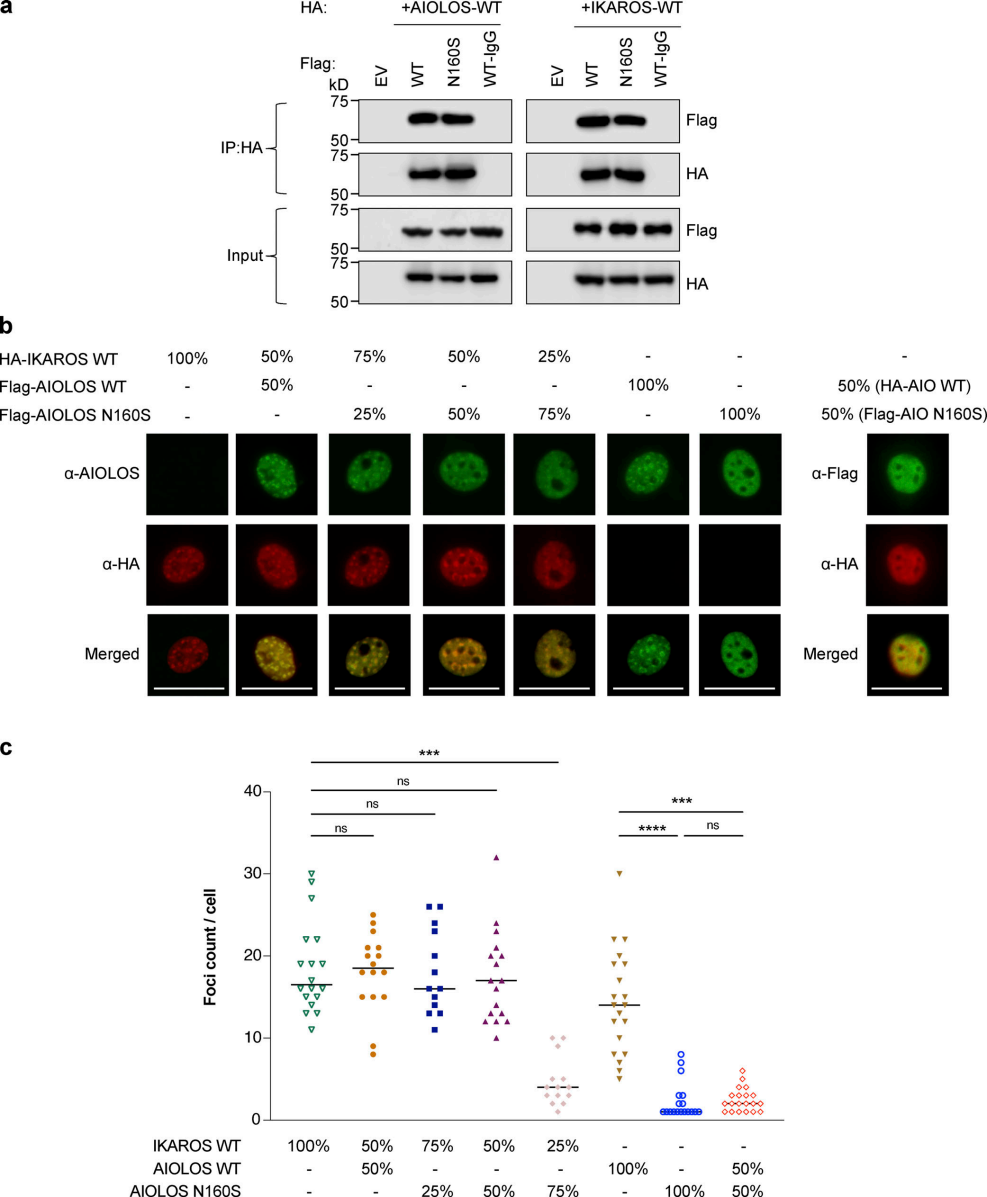
**The mutant AIOLOS N160S has normal hetero-/homodimerizations with AIOLOS WT and IKAROS WT.** **(a) **HEK293T cells were transfected with Flag-tagged AIOLOS WT or the mutant together with HA-tagged AIOLOS WT or IKAROS WT. EV indicates the empty vector control. IPs were performed using anti-HA or rabbit IgG antibodies. Western blot analysis of the IP samples with anti-HA and anti-Flag is shown. Input control indicates 5% of the total volumes of the whole cellular lysates used for IP reaction. Data shown are representative of three independent experiments. **(b) **NIH3T3 cells were transfected with the indicated ratio of WT IKAROS and/or AIOLOS WT or mutant expression vectors as indicated in the figure. Cells were visualized using an EVOS (20× or 40× objective) fluorescent cell imager. The scale bars indicate 25 µm. Data shown are representative of at least three independent experiments. **(c) **Graph shows the quantification of numbers of foci per cell in each group (three to six representative images from each experiment were selected). The horizontal lines indicate the median. Kruskal–Wallis test showed a statistically significant difference between the groups (P < 0.0001). Follow-up pairwise comparisons between groups (Dunn’s multiple comparisons test) are indicated (***, P < 0.001; ****, P < 0.0001).

AIOLOS homo- and heterodimerization with IKAROS, mediated through its C-terminal dimerization domain (Morgan et al., 1997), were also tested. Mutant AIOLOS N160S showed normal homodimerization to WT AIOLOS as well as heterodimerization to WT IKAROS (Fig. 3 a). To further test whether mutant AIOLOS N160S affected WT IKAROS functions when heterodimerized, both vectors were cotransfected into cell lines, and DNA binding and pericentromeric targeting were tested. The mutant AIOLOS N160S did not exert a dominant-negative effect over WT IKAROS for either DNA binding or pericentromeric targeting when the mutant and WT were expressed in an equal ratio (Fig. 2, c and d; and Fig. 3, b and c).

### B cell phenotypic analysis

**Figure 4.**
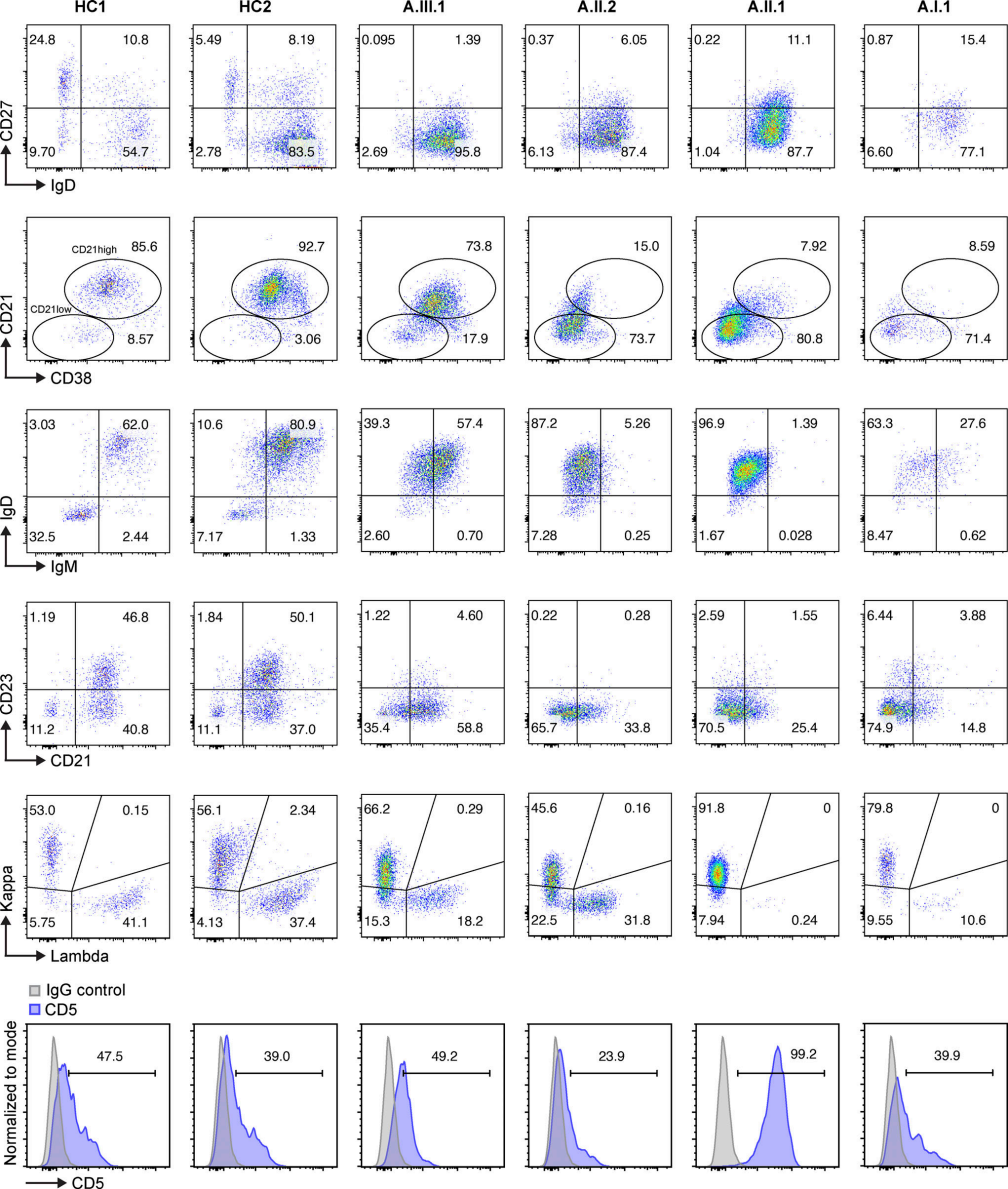
**B cell phenotypes in patients.** Multicolor flow cytometry analysis of subpopulations of B cells from healthy controls and patients are shown. B cells (CD3^−^CD19^+^) were gated from total lymphocytes, followed by analysis of B cell subpopulations. CD21^low^ B cells (CD19^+^CD21^−/low^CD38^−/low^), naive B cells (CD19^+^IgD^+^CD27^−^), and switched memory B cells (CD19^+^IgD^−^CD27^+^) are shown. Kappa, lambda, IgM, CD21, CD23, and CD5 expressions from total B cell population are shown. Representative data from two independent experiments are shown.

**Figure 5.**
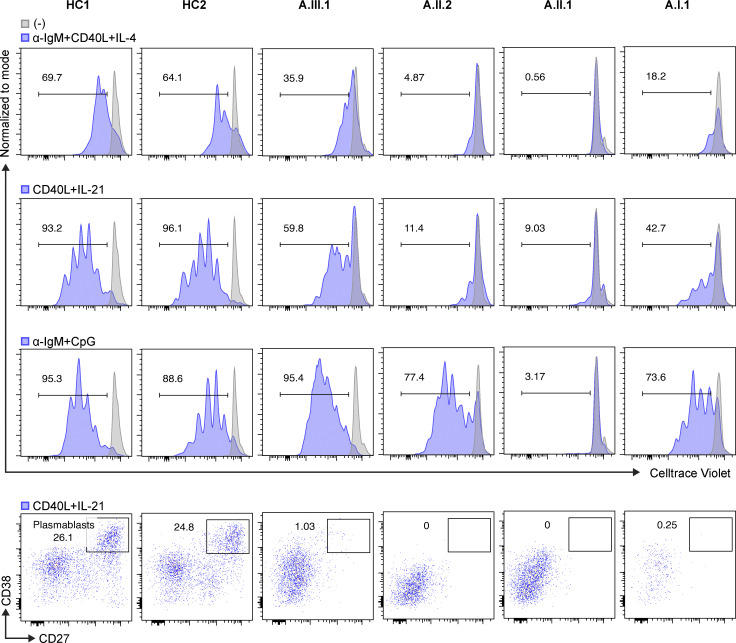
**Abnormal B cell proliferation and differentiation in patients.** Total PBMCs were stained with CellTrace Violet and stimulated with indicated agonists. After incubation for 4 d, cells were acquired and analyzed by flow cytometry and FlowJo software, respectively. The numbers indicate the frequency of proliferating B cells. CD40L- and IL-21–stimulated B cells were gated on CD27^+^ and CD38^+^ to see plasmablast differentiation. Representative data from two independent experiments are shown.

Although B cell deficiency is the most common immunological feature in patients with *IKZF1* mutations (Kuehn et al., 2021b), normal to high B cell numbers were observed in three of the family members, while they were low in the fourth (A.I.1; Table 1). Interestingly, all patients had increased CD21^low^ B cells with near absence of memory B cells, and all patients showed minimal CD23 expression compared with healthy controls (Fig. 4). B cell surface kappa and lambda light chain ratio was skewed in two patients (A.I.1 and A.III.1) with monoclonal B cells observed in one patient (A.II.1) who had atypical CLL. Further testing of CD5 expression on B cells showed that only the patient with CLL (A.II.1) had increased CD5 levels detected on B cells (Fig. 4). Decreased intensity of IgD, IgM, kappa, and lambda expression was also observed in all patients’ B cells. Extremely low serum IgG, IgA, and IgM levels were found in all patients at diagnosis. Given the severity of their hypogammaglobinemia, postvaccine antibody responses were not assessed. Further functional studies revealed that patients’ B cells exhibited significantly decreased proliferation to CD40L, in combination with either B cell receptor (BCR; anti-IgM) and IL-4 or IL-21 (Fig. 5). In contrast, BCR and CpG (TLR9 agonist) stimulation induced proliferation in three of the patients’ B cells but not the patient with CLL (A.II.1). Patients’ B cells also failed to differentiate into plasmablasts (CD27^+^CD38^+^) upon extrinsic CD40L and IL-21 stimulation, which is likely due to the low number of memory B cells as well as impaired CD40L/CD40 pathway signaling (Fig. 5). The increased CD21^low^ B cell population is likely associated with the reduced BCR (anti-IgM) response and impaired B cell activation upon CD40L stimulation (Isnardi et al., 2010).

**Table 1.** Laboratory results of immunological tests in patients with the heterozygous *IKZF3* mutation

ID	A.I.1 (Grandfather)	A.II.1 (Aunt)	A.II.2 (Father)	A.III.1 (Index Patient)	Reference range
Age (y) and sex	62, M	40, F	39, M	2, F	
Age at onset	15 y	5 y	1 y	4 mo	
**White blood cells (cells/μl)**					
Neutrophil	3,530	4,290	1,290 (L)	N.A.	Adult: 1,780–5,380
				1,380 (L)	6 mo–5 y: 1,500–8,500
Lymphocyte	1,270 (L)	3,510	1,830	N.A.	Adult: 1,320–3,570
				4,060	1–2 y: 3,000–9,500
Monocyte	660	750	460	N.A.	Adult: 300–820
				360	1–2 y: 300–850
Eosinophil	100	60	40	N.A.	Adult: 40–540
				60 (L)	1–2 y: 165–465
Basophils	80	100 (H)	40	N.A.	Adult: 10–80
				20	1 mo–5 y: 0–140
**Lymphocyte phenotyping**					
CD3 T (ALC; %)	1,015; 80	2,166; 62	1,448; 79	5,954; 69	Adult: 651–2,804; 55–88
				2,931; 72	21 mo: 2,100–6,200; 53–75
CD4 T (ALC; %)	631; 50	1,204; 34	525; 29	3,724 (H); 43	Adult: 370–1,336; 28–56
				1,803; 44	21 mo: 1,300–3,400; 32–51
CD8 T (ALC; %)	334; 26	769; 22	675; 37	1,821; 21	Adult: 185–1,024; 14–46
				865; 21	21 mo: 620–2,000; 14–30
CD4/CD8 ratio	1.89	1.6	0.78 (L)	2.04	Adult: 0.81–4
				2.08	21 mo: 1.2–6.2
CD4^+^CD45RA^+^ (%)	61 (H)	65 (H)	50	88	Adult: 14–57% of CD4^+^
				85	21 mo: 63–91
CD8^+^CD45RA^+^ (%)	19 (L)	61	23	94	Adult: 21–70% of CD8^+^
				90	21 mo: 71–98
CD4^+^CD45RA^+^CD31^+^: RTE (%)	67 (H)	68 (H)	55 (H)	82	Adult: 6.7–45% of CD4^+^
				79	21 mo: 40–100
CD4^+^CD45RA^−^CXCR5^+^: Tfh (%)	2.7 (L)	2.7 (L)	3.6 (L)	N.A.	Adult: 3.7–14% of CD4^+^
				1.8	21 mo: N.A
CD4^+^CD25^+^FOXP3^+^: T reg (%)	6.2	6.1	8.8	N.A.	Adult:4–10% of CD4^+^
				4.5	21 mo: N.A.
CD 20 B (ALC; %)	38 (L); 3	990 (H); 28 (H)	234; 13	1988, 23	Adult: 79–399; 3.8–18
				702, 17	21 mo: 720–2,600; 16–35
CD20^+^CD27^+^IgM^−^ or IgD^−^ (%; switched memory)	0 (L)	2.5 (L)	0 (L)	0.7	Adult: 4.3–20% of CD19^+^
				0 (L)	21 mo: 0.1–1.9
CD19^+^CD38^++^CD24^−^ (%; plasmablast)	0 (L)	0 (L)	0 (L)	N.A.	Adult: 0.3–3.6 of CD19^+^
				0	21 mo: N.A
NK (ALC; %)	222; 18	362;10	154; 8	587, 7	Adult: 126–841; 7.3–33
				414, 10	21 mo: 180–920; 3–15
NKT (ALC; %)	61; 5	249; 7	613 (H); 34 (H)	N.A.	Adult: 56–448; 2.1–18
				28, 0.7	21 mo: N.A.
**Immunoglobulins (mg/dl)**					
IgG	1,163^a^	914^a^	1,420^a^	4 mo: 85 (L)	Adult: 540–1,822
				25 mo: 916^a^	4 mo: 311–664
IgA	8 (L)	<5 (L)	10 (L)	4 mo: 15	Adult: 101–645
				25 mo: <5 (L)	4 mo: 0–42
IgM	<5 (L)	<5 (L)	<5 (L)	4 mo: 57	Adult: 22–240
				25 mo: <5 (L)	4 mo: 0–127

H and L indicate values above and below the age-matched normal range, respectively. Adult reference values are from in-house, and pediatric reference values are from Children’s Hospital Colorado and the published ranges (Nathan and Oski, 1987). For the pediatric index patient A.III.1, laboratory tests at two different time points (21 mo [upper line; 4 mo for immunoglobulins] and 25 mo [bottom line]) are shown. ALC, absolute lymphocyte count; N.A., not applicable; RTE, recent thymic emigrant; T reg, regulatory T cell.

a Patient on intravenous immunoglobulin.

### T cell phenotypic analysis

**Figure 6.**
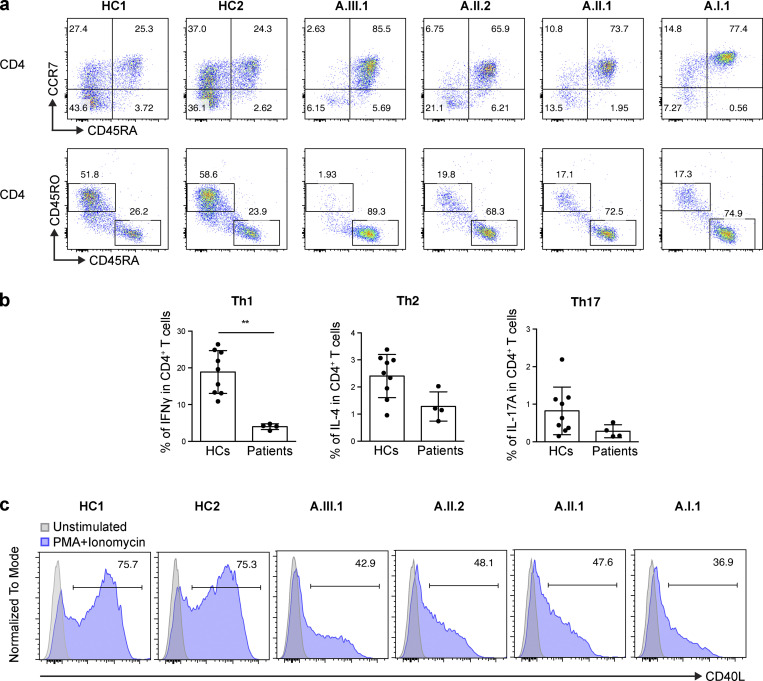
**T cell phenotypes in patients.** **(a) **CD3^+^ T cells were gated from total lymphocytes, followed by analysis of CD4 T cell subpopulations. CD45RA and CCR7 expression patterns were used to identify naive T cells (CD45RA^+^CCR7^+^), central memory T cells (CD45RA^−^CCR7^+^), effector memory T cells (CD45RA^−^CCR7^−^), and terminal effector memory T cells (CD45RA^+^CCR7^−^). **(b) **The percentages of IFNγ (Th1), IL-4 (Th2), and IL-17A (Th17) from CD3^+^CD4^+^ T cells are shown. Data are mean ± SD from nine different healthy controls and four patients. Each dot in the patient group represents the mean value from two replicates. Mann–Whitney *U* test, **, P < 0.01. **(c) **CD40L expression on CD4 T cells after PMA and ionomycin stimulation. Data shown are representative of two independent experiments.

CD3^+^ T cell numbers were normal, with markedly increased recent thymic CD4 T cell emigrants (CD3^+^CD4^+^CD45RA^+^CD31^+^) observed in A.I.1, A.II.1, and A.II.2 (Table 1). While naive CD4 T cells were also increased, memory T cells, lineage-committed T helper 1 (Th1; CD4^+^IFNγ^+^) cells, and T follicular helper (Tfh; CD4^+^CXCR5^+^CD45RA^−^) cells were markedly reduced (Table 1 and Fig. 6, a and b).

**Figure S1.**
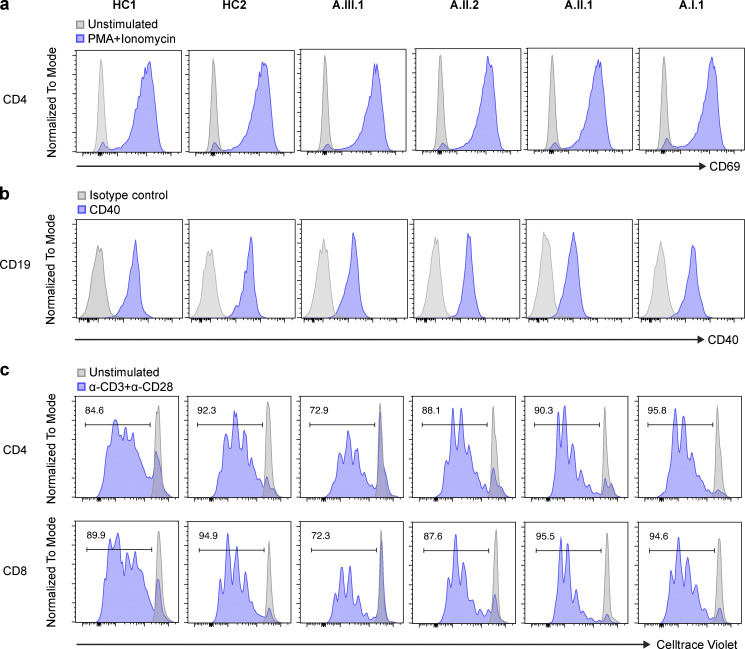
**CD40 expression on B cells and TCR-induced T cell proliferation.** **(a) **Enriched CD4 T cells were stimulated with PMA and ionomycin for 20–24 h, and up-regulation of CD69 was tested on the cells. **(b) **Total PBMCs were stained with CD19 and CD40, and CD40 expression on B cells was analyzed by flow cytometry. Gray peak represents an isotype control. **(c) **CellTrace Violet–stained PBMCs were stimulated with anti-CD3 and anti-CD28 for 4 d. Proliferation histograms for stimulated (blue) and unstimulated (gray) cells are shown. The numbers indicate the frequency of proliferating T cells. Data shown are representative of two independent experiments.

CD40L expression on activated T cells and its interaction with CD40 plays an essential role in both T cell–mediated immune responses and B cell development and class switching (Grewal and Flavell, 1998; van Kooten and Banchereau, 2000). CD40L deficiency is associated with low IgG and IgA with normal/elevated IgM and normal B cell numbers, as well as increased susceptibility to PJP and recurrent bacterial infections, among other features (França et al., 2019; Levy et al., 1997; Notarangelo et al., 2006). Because of the overlapping clinical features between our AIOLOS patients and CD40L/CD40 pathway–deficient patients (e.g., hypogammaglobulinemia and PJP), we examined CD40L expression on T cells. Following PMA and ionomycin stimulation, adequate cell activation was observed as evaluated by CD69 expression, but CD40L expression was markedly decreased in all patients tested (Figs. 6 c and S1 a). Of note, TCR-induced T cell proliferation was largely normal, as was CD40 expression on B cells (Fig. S1, b and c). These results suggest that impaired CD40L up-regulation in T cells and defective CD40 signaling in B cells likely contribute to the B cell differentiation defects as well as PJP susceptibility in the patients. Moreover, the fact that AIOLOS N160S patients present with low serum and cell surface expression of IgM, and that their B cells do not respond to extrinsic CD40L stimulation, may indicate that their IgM/humoral defect has an intrinsic B cell component that precedes CD40L regulation (Table 1 and Fig. 4).

### RNA sequencing (RNA-seq)

**Figure 7.**
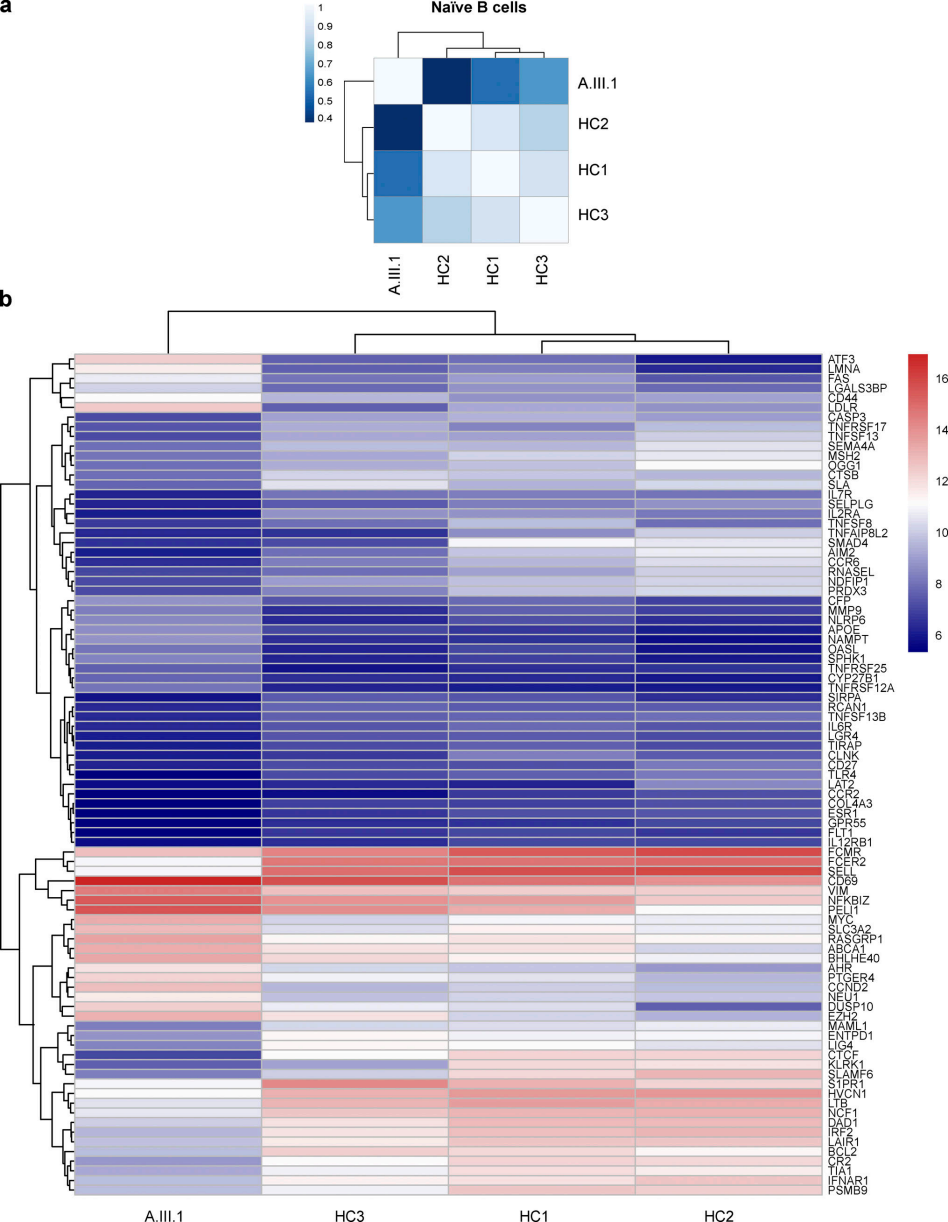
**RNA-seq analyses for naive B cells.** **(a) **Pearson correlation analyses of the count matrices. The scale bar represents the range of the correlation coefficients (*r*). **(b) **Heatmaps for 87 differentially expressed immune-related genes in naive B cells. Red is expressed at high levels and blue is expressed at low levels.

To further investigate the functional impact of AIOLOS N160S mutation in transcriptional regulation, we performed RNA-seq on naive B cells (due to the limited sample availability, only performed in the index patient) and T cell blasts. Different gene expression signatures between healthy controls and the index patient B cell results were found by correlation and heatmap analyses (Fig. 7). Among the 997 genes that were differentially expressed in the index patient, we specifically evaluated 87 immune-related genes. *CR2* (CD21) and *FCER2* (CD23) were reduced at the RNA level (Fig. 7 b), correlating with the reduced protein expression observed in the patients’ B cells (Fig. 4). Several other genes known to be associated with B cell development and differentiation, including *TNFRSF17* (BCMA), *TNFSF13* (APRIL), and *TNFSF13B* (BAFF) were also down-regulated in the patient sample. Bcl-2 expression, which has been shown to be regulated by Aiolos (Romero et al., 1999), was also reduced in this patient (Fig. 7 b). When BCL2 protein expression was tested in the patients’ naive B cells by flow cytometric analysis, slightly low to normal levels were observed in three patients, while levels were slightly elevated in the fourth (data not shown). This apparent discrepancy between the RNA-seq and protein expression data could be explained by posttranscriptional and posttranslational modifications or protein degradation, among other reasons.

**Figure S2.**
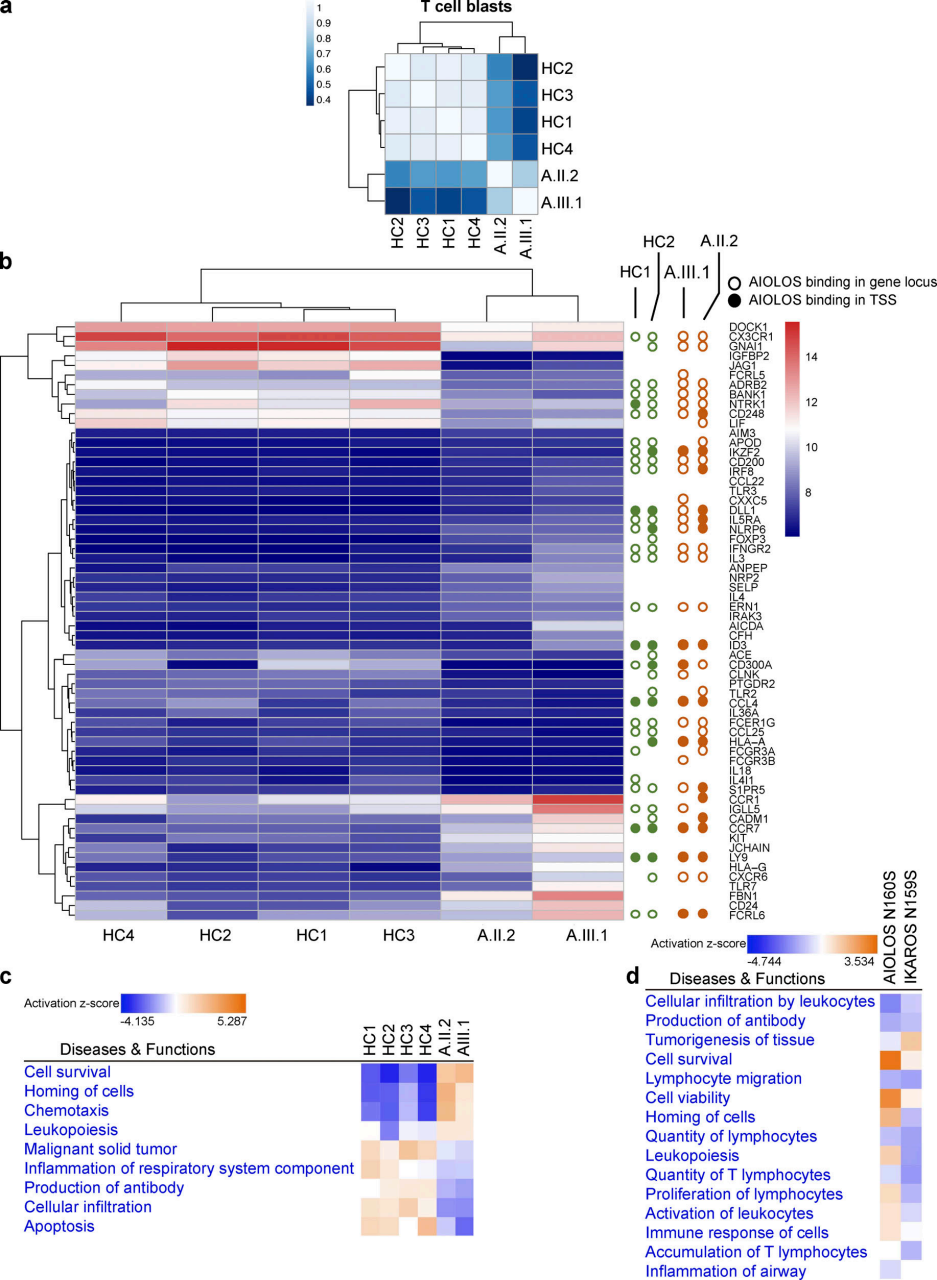
**RNA-seq analyses.** **(a) **Pearson correlation analyses of the count matrices for T cell blasts. The scale bar represents the range of the correlation coefficients (*r*). **(b) **Heatmaps for 62 differentially expressed immune-related genes in T cell blasts. Red is expressed at high levels and blue is expressed at low levels. Open circles indicate AIOLOS binding in the gene loci (upstream/downstream/exonic/intronic), and filled circles indicate AIOLOS binding in TSS from the ChIP-seq data performed in T cells. Green and orange colors indicate healthy donor controls and patients, respectively. **(c) **IPA Diseases and Biofunctions analysis for T cell blasts from the patients with the AIOLOS N160S. **(d) **Comparison of RNA-seq data analysis for AIOLOS N160S and IKAROS N159S studies performed in T cell blasts.

When the RNA-seq was focused on TCR-induced T cell blasts, 360 genes were differentially expressed in the AIOLOS N160S patient cells (A.II.2 and A.III.1), 62 of which are associated with immune cell signaling (Fig. S2, a and b). Ingenuity pathway analysis (IPA) Diseases and Functions evaluation showed that the differentially regulated genes encoded proteins that were associated with cellular infiltration, malignant solid tumors, leukopoiesis, homing of cells, chemotaxis, cell survival, antibody production, and inflammation of respiratory system components, among others (Fig. S2 c and Table S2). These dysregulated gene expression patterns in T and B lymphocytes likely contribute to the immunological and clinical features in AIOLOS N160S patients.

We also compared the RNA-seq data between AIOLOS N160S and IKAROS N159S dominant-negative patients, both sharing similar clinical (infections and PJP) and immunological (increased naive and decreased memory T cells) features (Boutboul et al., 2018). RNA-seq of T cell blasts revealed that among 807 differently expressed genes, 104 were common to patients with either AIOLOS N160S or IKAROS N159S mutations. The direction of the fold-changes was consistent for the majority of genes (94/104). IPA comparison of the biological functions in AIOLOS N160S and IKAROS N159S patients showed that while IKAROS and AIOLOS have many similar effects on the immune system, they also have selected differential and nonredundant effects (Fig. S2 d and Table S2). IPA Diseases and Functions analysis showed that the differentially expressed genes encoded proteins that were associated with cellular infiltration by leukocytes, lymphocyte migration, cell survival, and antibody production, among others (Fig. S2 d and Table S2). These shared gene expression patterns may explain the similar clinical and immunological phenotypes between the two diseases.

**Figure S3.**
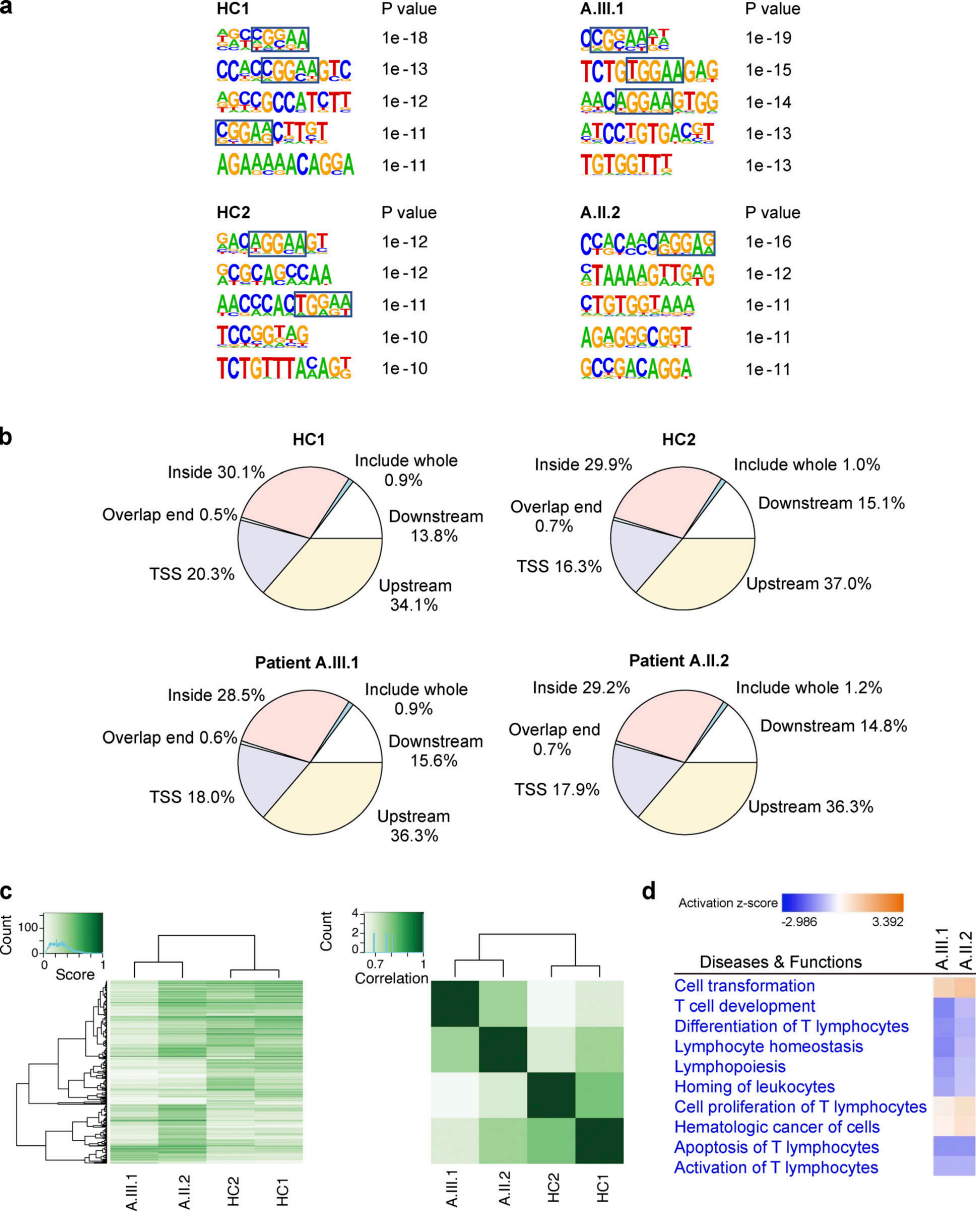
**ChIP-seq analyses.** **(a) **The top significant DNA-binding motifs with P values for healthy controls and two patients. The top 1,000 highly enriched peaks bound by representative ChIP–seq samples are shown. **(b) **Genome-wide distribution of AIOLOS ChIP-seq peaks in T cells. **(c) **A binding affinity heatmap and a correlation heatmap with clustering of the ChIP-seq samples are shown. **(d) **IPA Diseases and Biofunctions analysis of ChIP-seq data from the patients with AIOLOS N160S.

Chromatin immunoprecipitation (IP) followed by sequencing (ChIP-seq) analysis from T cells from A.II.2 and A.III.1 and two healthy controls was also evaluated to further investigate the functional impact of AIOLOS N160S mutation in transcription regulation. No significant changes were observed in the conserved DNA binding motifs (NGGAA) or the location of the binding sites between healthy controls and the patients (Fig. S3, a and b). To determine the correlation between ChIP-seq and RNA-seq data, we compared the RNA-seq differentially expressed genes with the ChIP-seq–determined AIOLOS binding sites (upstream/downstream/exonic/intronic vs. transcription start sites [TSS]). No remarkable differences were detected between healthy controls and patients when this analysis was done either (Fig. S2 b). While there were no differences in DNA binding motifs, heatmap analysis of the differentially occupied regions did show differential binding patterns between patients and healthy controls (Fig. S3 c). IPA Diseases and Functions analysis showed that the genes with higher differential occupation in patients compared with controls were associated with proteins involved in proliferation and transformation, whereas those with lower differential occupation were associated with proteins involved in lymphocyte homeostasis and T cell development and differentiation (Fig. S3 d and Table S2).

### T and B phenotypes in Ikzf3^N159S^ mutant mice

**Figure S4.**
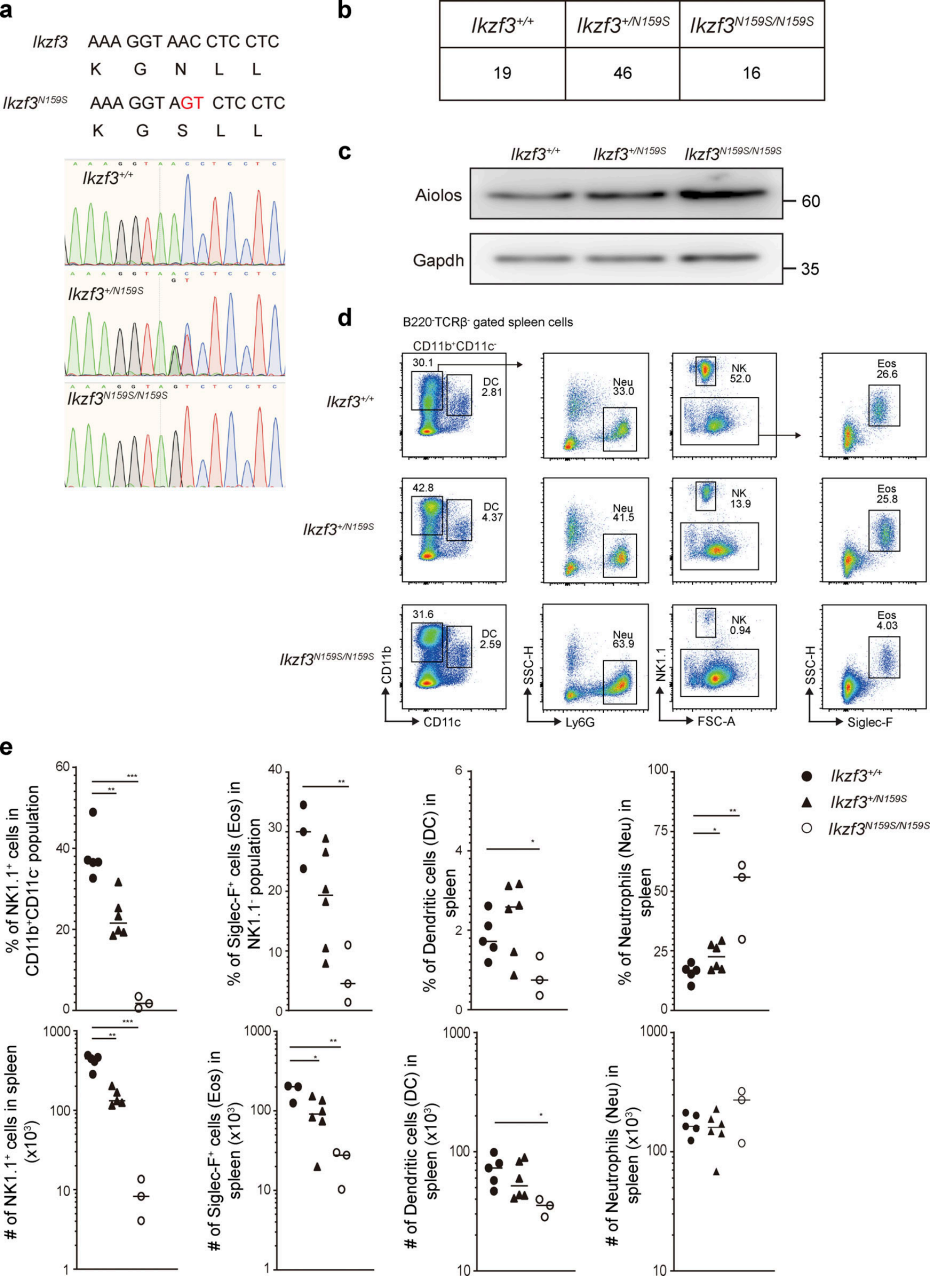
**Generation of *Ikzf3^N159S^* mouse strain and nonlymphoid immune cells in mice.** **(a) **Sanger sequencing of the surrounding region in the *Ikzf3* N159S from mice with the indicated genotype. Upper panel shows the designed mutation for the *Ikzf3^N159S^*. **(b) **Table showing the number of 1-wk-old mice with the indicated genotype that were born by heterozygous-to-heterozygous crossing. **(c) **Immunoblot showing Aiolos protein expression in total thymocytes. Gapdh was used as a loading control. Data are representative of three independent experiments. **(d) **Total splenocytes were gated on the B220^−^TCRβ^−^ population, and then analyzed dendritic cells (DC; CD11b^+^CD11c^+^), neutrophils (Neu; CD11b^+^CD11c^−^ Ly6G^+^), NK cells (CD11b^+^CD11c^−^NK1.1^+^), and eosinophils (Eos; CD11b^+^CD11c^−^NK1.1^−^Siglec-F^+^) as shown in the figure. FSC, forward scatter. **(e) **Graphs show the frequency (top) and absolute numbers (bottom) of indicated populations from WT *Ikzf3^+/+^* littermates (filled circle), *Ikzf3^+/N159S^* heterozygous mice (filled triangle), and *Ikzf3^N159S/N159S^* homozygous mice (open circle). Horizontal lines indicate the median values of at least three different mice (2–3 mo old) per group. Statistically significant differences (Student’s unpaired, two-tailed *t *test) between the groups are shown. *, P < 0.05; **, P < 0.01; ***, P < 0.001.

**Figure 8.**
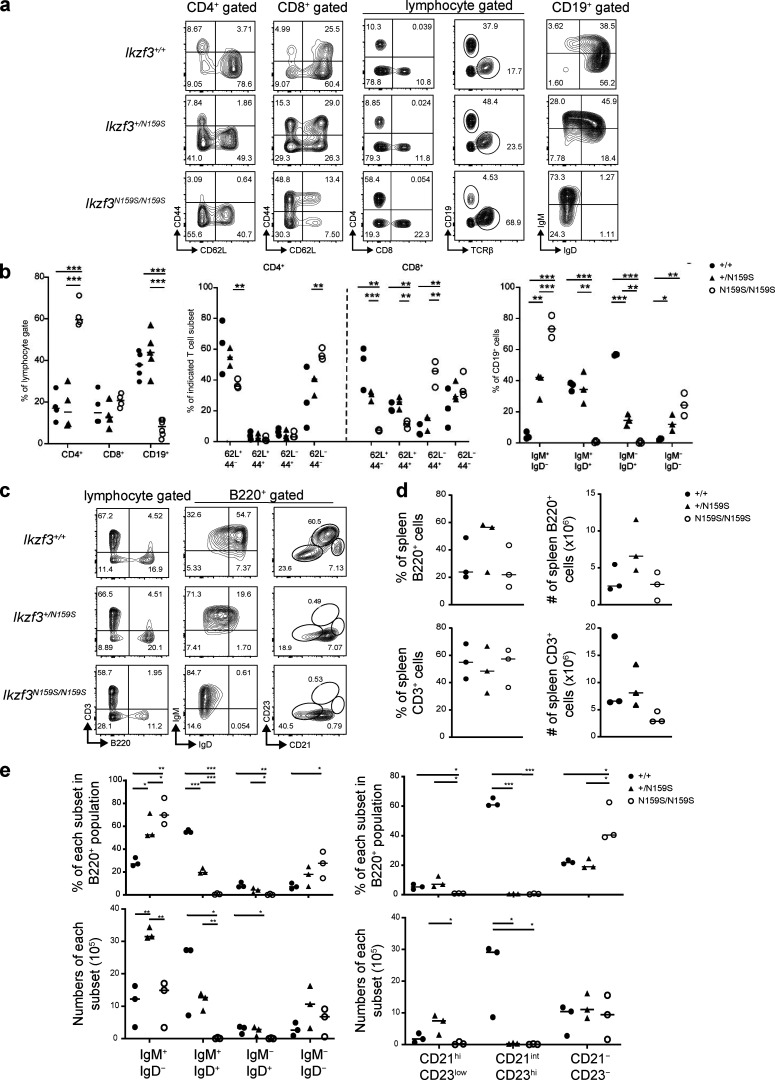
**Lymphocyte phenotypes in *Ikzf3^N159S^* mice.** **(a) **FACS analysis of peripheral T and B cell population using the indicated markers. CD44 and CD62L were used to determine naive (CD62L^+^CD44^−^), central memory (CD62L ^+^CD44^+^), and effector memory (CD62L^−^CD44^+^) T cell subsets. IgM and IgD expressions on CD19^+^ peripheral blood B cells are shown. **(b) **Graphs show the frequency of indicated populations. Each dot represents an individual mouse. The horizontal lines indicate the median values of at least three different mice per group. **(c) **FACS analysis of splenic B cells population stained for IgM, IgD, CD21, and CD23. **(d) **Frequency (left) and absolute numbers (right) of splenic B and T cell populations from the WT *Ikzf3^+/+^* littermates (filled circle), *Ikzf3^+/N159S^* heterozygous (filled triangle), and *Ikzf3^N159S/N159S^* homozygous (open circle) mice. Horizontal lines indicate the median. **(e) **Graphs show the frequency of indicated populations from B220^+^ cells (top) and absolute numbers (bottom) of each population. Horizontal lines indicate the median values of three different mice per group. Statistically significant differences (Student’s unpaired, two-tailed *t *test) between the groups are shown. *, P < 0.05; **, P < 0.01; ***, P < 0.001.

**Figure 9.**
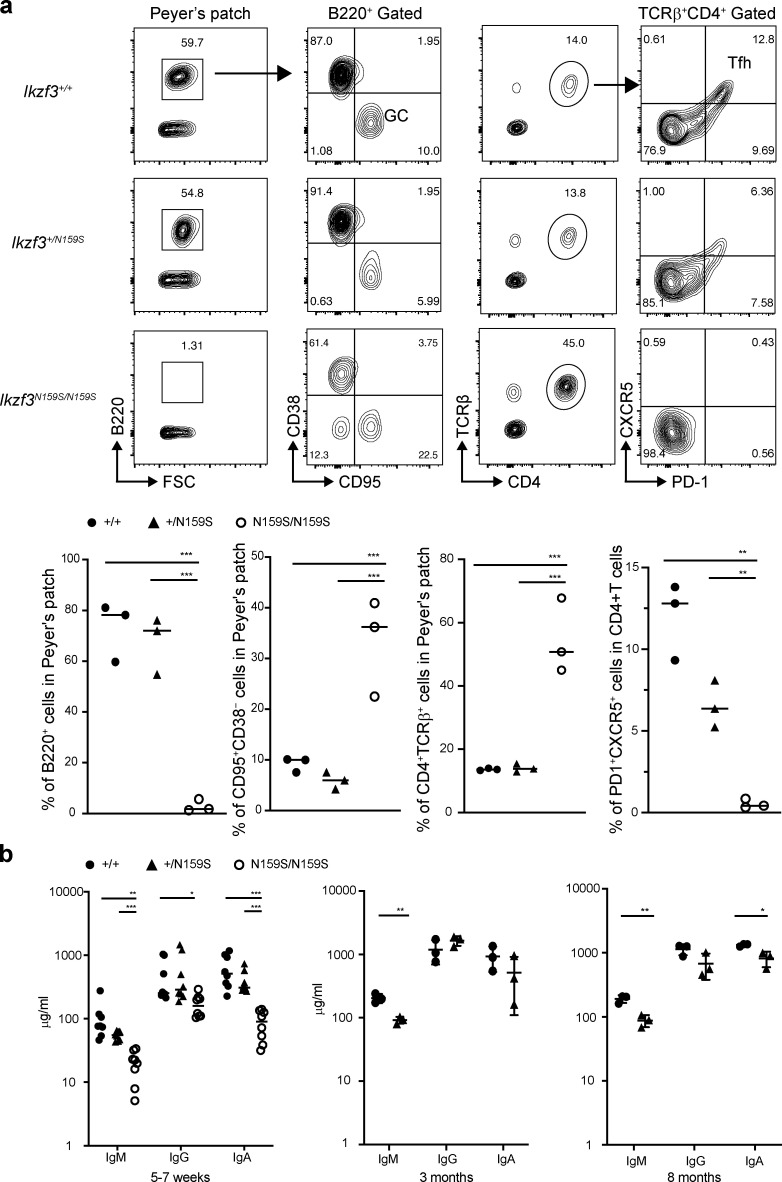
**Impaired differentiation of germinal center (GC) B cells and Tfh cells in *Ikzf3^N159S^* mice.** **(a) **Flow cytometry profiles of lymphocytes in Peyer’s patch. B220^+^ population was nearly absent in *Ikzf3^N159S/N159S^* mice, with increased frequency of CD95^+^CD38^−^ GC B cells. In the CD4^+^ T cell population, CXCR5^+^PD-1^+^ Tfh was not observed in *Ikzf3^N159S/N159S^* mice. Graphs show the frequency of indicated populations. Horizontal lines indicate the median values of three different mice per group. WT *Ikzf3^+/+^* littermates (filled circle), *Ikzf3^+/N159S^* heterozygous mouse (filled triangle), and *Ikzf3^N159S/N159S^* homozygous mouse (open circle) data are shown. FSC, forward scatter. **(b) **Concentrations of serum IgM, IgG, and IgA of mice (*n* = 3–8) with each genotype at 5–7 wk, 3 mo, and 8 mo old are shown. Horizontal lines indicate the median. Statistically significant differences (Student’s unpaired, two-tailed *t *test) between the groups are shown. *, P < 0.05; **, P < 0.01; ***, P < 0.001.

A knock-in *Ikzf3*/Aiolos^N159S^ mouse strain carrying the N159S mutation (corresponding to the human AIOLOS N160S mutation) was generated by genome editing to further validate the effect of this missense mutation on T and B cell development and differentiation (Fig. S4 a). Homozygous mutant mice *Ikzf3*^N159S/N159S^ were produced with the expected Mendelian frequency, and Aiolos expression in heterozygous mutant mice was comparable to that of WT littermates and slightly high in homozygous mice (Fig. S4, b and c). While the homozygous *Ikzf3*^N159S/N159S^ mice showed a significant increase in T cells and a decrease in B cell frequencies in peripheral blood, modestly increased T and B cell populations were observed in the *Ikzf3*^+/N159S^ heterozygous mice compared with the WT littermates. CD4:CD8 ratio and naive vs. memory phenotypes were comparable between WT and heterozygous mice, whereas homozygous mice showed an increase in CD4^+^ T cell population and a decrease in naive (CD62L^+^CD44^−^) T cells (Fig. 8, a and b). A defect in B cell development was notable both in hetero- and homozygous mice (Fig. 8). Mature follicular B cells (IgM^low^IgD^hi^ or CD21^int^CD23^hi^) were significantly decreased in the spleen in both hetero- and homozygous mice (Fig. 8, c and e). IgM/IgD expression profiles were also impaired by the *Ikzf3*^N159S^ variant in both hetero- and homozygous mice, and IgD expression was abrogated in *Ikzf3*^N159S/N159S^ mice (Fig. 8). When Peyer’s patches were analyzed, homozygous mice showed nearly absent Tfh cells (CXCR5^+^PD-1^+^) and B220^+^ B cell lineages with increased germinal B cell (CD38^−^CD95^+^) frequency in the residual small B220^+^ population (Fig. 9 a). Serum IgG, IgA, and IgM levels were also significantly decreased in homozygous but not heterozygous mice when analyzed at 5–7 wk of life. However, when heterozygous mice were evaluated at 3 and 8 mo of age, reduced IgM (3 mo) and reduced IgM and IgA (8 mo) were demonstrated (Fig. 9 b). When absolute numbers of other cell lineages were evaluated, heterozygous and homozygous mice showed results comparable to WT for neutrophils and dendritic cells (although minimally decreased in homozygous), while eosinophils and NK cells were decreased in both mice (Fig. S4, d and e).

In short, the mouse data demonstrated that the mutant Aiolos N159S particularly alters T cell differentiation and B cell maturation and function, resembling certain immunological phenotypes observed in patients carrying the AIOLOS N160S mutation.

## Discussion

Here we describe four patients in a multigenerational family carrying a novel heterozygous variant in *IKZF3* (NM_012481:c.479 A>G, p.N160S) associated with T and B cell developmental and functional defects, CID, and PJP, as well as CLL (in one patient). The disease segregates in an autosomal dominant fashion and appears to be fully immunologically and clinically penetrant (as no immunocompetent/asymptomatic carriers were detected), while clinical expressivity may vary. We demonstrated that mutant AIOLOS N160S has both impaired DNA binding to its consensus sequence and pericentromeric localization. This mutant exerted a dominant-negative effect on WT AIOLOS but not on WT IKAROS. Patients with the N160S mutation presented with disturbances affecting lymphoid lineages (following AIOLOS expression pattern), manifesting as abnormal T and B cell maturation and function accompanied by severe hypogammaglobulinemia, opportunistic infections, and likely increased hematologic malignancy susceptibility. Interestingly, AIOLOS N160S is the site homologous to IKAROS N159S, with patients carrying this particular *IKZF1* mutation also displaying abnormal T and B cell phenotypes, opportunistic infections (i.e., PJP), and hematologic malignancy susceptibility (Boutboul et al., 2018). Immunological and clinical features between these two diseases show overlapping characteristics, except for the B cell numbers, which were observed to be nearly absent in IKAROS N159S patients. T cell numbers are normal to elevated in both conditions, with markedly increased naive CD4 T cells and impaired Th polarization and T cell function also observed. Moreover, PJP is a hallmark in both IKAROS N159S– and AIOLOS N160S–associated diseases. Mechanistically, while IKAROS N159S exerts a dominant-negative effect over both WT IKAROS and WT AIOLOS (Boutboul et al., 2018), AIOLOS N160S shows a dominant-negative effect over WT AIOLOS but not WT IKAROS function. This discrepant mechanistic effect between the two homologous variants may be supported by mutation-dependent as well as mutation-independent features: e.g., protein modeling of AIOLOS N160S ZF2 (where the mutation is located and a critical domain for DNA binding) predicts markedly increased protein flexibility, whereas IKAROS N159S ZF2 is predicted to have markedly increased protein rigidity, both effects depending on the mutations as well as AIOLOS/IKAROS differences in the ZF2/ZF3 linker areas (not shown). Taken together, these data suggest that AIOLOS itself likely plays a critical and independent role in normal T and B cell differentiation and function, as well as in protective immunity to PJP.

All four AIOLOS N160S patients showed a particular B cell maturation abnormality characterized by decreased class-switched memory B cells, plasmablasts, and CD23 expression (likely contributing to the hypogammaglobulinemia with recurrent sinopulmonary infections), as well as increased CD21^low^ B cells (Table1 and Fig. 4). *Ikzf*3*^N159S^* hetero- and/or homozygous mice showed different degrees of similarly impaired B cell maturation and functional defects, including absent follicular B cells (CD21^int^CD23^hi^) and low serum immunoglobulins. The variation between the two models (hetero- vs. homozygous) likely represents an *Ikzf*3 gene dosage effect (Figs. 8 and 9). Taken together, these human and murine findings demonstrate that AIOLOS plays a crucial role in normal B cell maturation and function.

Expansion of CD21^low^ B cells in the peripheral blood has been reported in patients with CVID with autoimmunity and immune dysregulation (Ahn and Cunningham-Rundles, 2009; Nichols et al., 2015; Warnatz et al., 2002). CD21^low^ B cells in CVID patients have a poor response to BCR-mediated signaling, including after costimulation with CD40L; increased apoptosis; and subnormal proliferation in response to TLR9 stimulation (Isnardi et al., 2010; Rakhmanov et al., 2009). In agreement with previous reports, we found poor B cell proliferation after CD40L stimulation and reduced to normal proliferation following TLR9 and BCR costimulation. Although all four patients had increased CD21^low^ B cells, the index patient A.III.1 had the lowest increase (∼18%) compared with the other affected family members (>70%). However, the impairment in CD40L-induced B cell proliferation and plasmablast differentiation was consistent, suggesting that conventional B cells and CD21^low^ B cells are similarly dysfunctional in AIOLOS patients (Fig. 5). Remarkably, while IgM intensity on CD21^low^ B cells has been reported to be increased in CVID patients, IgM expression was low to absent in all four patients with the AIOLOS N160S mutation. This suggests that the CD21^low^ B cells in these patients are distinct from CD21^low^ B cells reported in CVID patients (Isnardi et al., 2010; Rakhmanov et al., 2009), further implying a unique intrinsic B cell defect (involving all major immunoglobulin isotypes, including IgM) linked to AIOLOS N160S. In Aiolos-null mice (*Ikzf3*^−/−^), while germinal centers, mature follicular B cells, and serum immunoglobulin levels were increased, marginal-zone B cells were virtually absent (Cariappa et al., 2001; Wang et al., 1998). This was associated with down-regulation of CD21 expression in follicular B cells at both protein and mRNA levels (Cariappa et al., 2001). Unlike *Ikzf3*^−/−^ mice, *Ikzf3*^N159S/ N159S^ mice showed nearly absent follicular B cells (CD21^int^CD23^hi^) and germinal center B cells (CD38^−^CD95^+^) and low serum immunoglobulins (Figs. 8 and 9). Of note, both CD21 and CD23 expression levels were reduced in spleen B cells from hetero- and homozygous *Ikzf3*^N159S^ mice. Patients with AIOLOS N160S mutation also showed markedly reduced CD21, along with low CD23 expression, at both protein and mRNA levels (Figs. 4 and 7 b). These results suggest that AIOLOS/Aiolos plays an important role in regulation of CD21 and CD23 expression on B cells, and decreased CD21 and CD23 expression represents a potentially useful and sensitive screen for similar patients (e.g., was detected in all AIOLOS N160S affected individuals, but not in healthy controls), although its specificity (e.g., prevalence in all other primary immunodeficiency/inborn errors of immunity) has yet to be formally determined.

**Figure S5.**
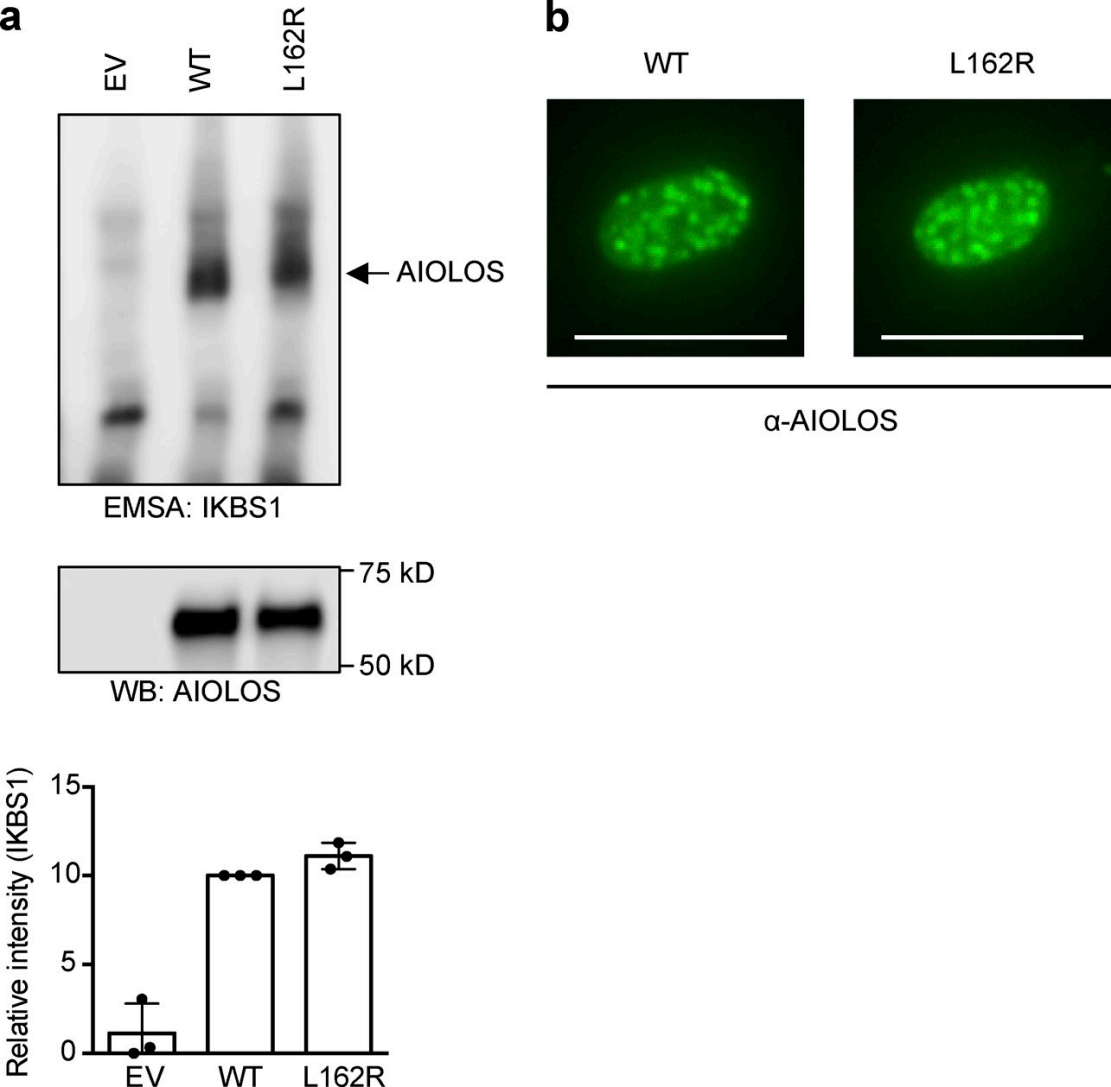
**DNA binding and pericentromeric targeting of AIOLOS L162R.** **(a) **EMSA was performed using nuclear extracts from HEK293T cells transfected with Flag-tagged AIOLOS WT or the mutant L162R. EV indicates the empty vector control. Nuclear extracts were subjected to Western blotting to test AIOLOS expression. Densitometry was performed to quantify AIOLOS binding to the IKBS1 probe, and the values were normalized to AIOLOS protein levels. The bar graph shows the relative IKBS binding levels normalized to WT samples. Data are expressed as mean + SD (*n* = 3). WB, Western blot. **(b) **NIH3T3 cells were transfected with Flag-tagged AIOLOS WT or the mutant L162R expression vector. Cells were stained with an anti-AIOLOS antibody, followed by Alexa Fluor 488–conjugated secondary antibodies. Cells were visualized by using an EVOS fluorescence microscope (20× objective). The scale bars indicate 25 µm. Data shown are representative of three independent experiments.

When focused on CLL susceptibility, reduced CD21 expression has been reported on CLL B cells (particularly in unmutated *IGHV* CLL) and has also correlated with disease stage and poor prognosis (Nichols et al., 2015). A.II.1 was diagnosed with CLL at the age of 30, with a CD5^+^, CD23^−^ (atypical for CLL) population of monoclonal B cells without *IGHV* mutation or 11;14 translocation. Bone marrow analysis revealed a 10% infiltration of cells positive for CD5, CD25, CD19, CD20, CD22, CD45, and HLA-DR; the clonal population was identified as IgVH1-2. Karyotype was normal 46XX. Interestingly, a second family member (A.I.1) has a skewed kappa/lambda B cell population (∼8:1 ratio), suggesting that some type of B cell clonal disease is emerging. Since 2008, an association between CLL and *IKZF3*/AIOLOS has been established. Two different mechanisms have been described to connect them: (a) AIOLOS reduced expression levels through epigenetic regulation (of note, AIOLOS expression levels in our patients carrying the N160S was comparable to healthy controls; Fig. 1 c; Billot et al., 2011; Duhamel et al., 2008) and (b) a specific recurrent somatic mutation affecting AIOLOS ZF2 (Landau et al., 2015). The latter study evaluating putative cancer-associated genes in CLL showed that 2% of CLL patients (11/538) carried somatic mutations in *IKZF3/*AIOLOS (Landau et al., 2015). Interestingly, all 11 patients had the same L162R variant mapping to the ZF2 DNA binding domain (the same ZF affected by the N160S variant in the family in this report), suggesting that L162R, and probably ZF2, is a hotspot and a putative driver of CLL. When we tested mutant L162R for its DNA-binding and PC-HC–targeting abilities, no defect in either function was detected (Fig. S5). Similar to our data, no defect of the PC-HC targeting was observed in B cells from mice with the L161R mutation (corresponding to human AIOLOS L162R), and increased density of nuclear dots was detected in the homozygous mice when compared with WT mice (Lazarian et al., 2021). Mice with the L162R mutation showed enhanced BCR/NF-κB signaling, abnormal B cell differentiation, enhanced germinal center formation, and late development of a CLL-like disease at age 18–24 mo. This B cell proliferative disease phenotype was also observed in elderly Aiolos-knockout mice (Wang et al., 1998). Although we have not seen CLL (or any other hematologic malignancy) in mice carrying the Aiolos N159S mutation through the age of 8 mo, a follow-up extending beyond the age of 2 yr will be required to definitively address this issue. While the AIOLOS-CLL association is supported by previous published data, it has only been diagnosed in one quarter of the affected patients, and therefore caution is warranted: it remains speculative at this point awaiting further studies and larger cohorts of affected individuals are identified.

In addition to CLL, patient A.II.1 was also diagnosed with metastatic melanoma at the age of 35. While we cannot completely rule out the AIOLOS-melanoma association, this connection, if causal, seems unlikely to be direct, as AIOLOS has a hematopoietic-exclusive/lymphoid-restricted expression pattern. Interestingly, Cereblon/CRBN-deficient T cells resulting in AIOLOS reduced degradation/increased expression, exert a more efficient antigen-specific cytosolic activity against melanoma cells both in vitro and in vivo (Hesterberg et al., 2020). Thus, it could be hypothesized that reduced AIOLOS expression or function might result in increased melanoma severity, poor immunosurveillance, and poor clinical outcome, although not increased susceptibility to cancer, per se.

We used ChIP-seq as a complementary approach to RNA-seq to study AIOLOS-specific transcription regulatory pathways. While the cell source between the two methods was not identical (RNA-seq, T cell blasts; ChIP-seq, T cells), the ChIP-seq data did show differences in the occupied regions between the healthy controls and the patients for genes in related pathways previously identified by RNA-seq (e.g., RNA-seq: apoptosis and cell survival, Fig. S2 c; ChIP-seq: apoptosis of T lymphocytes, Fig. S3 d). Moreover, the ChIP-seq data also validated the RNA-seq data by identifying the differentially expressed genes as targets of AIOLOS (Fig. S2 b).

Genome-wide association studies have shown that particular single nucleotide polymorphisms in *IKZF3* are associated with autoimmune diseases (Cai et al., 2014; Kurreeman et al., 2012; Lessard et al., 2012), suggesting that normal AIOLOS function is essential to maintain immune homeostasis. Several studies have shown that expansion of CD21^low^ B cells is frequently observed in CVID patients developing autoimmunity and immune dysregulation (Ahn and Cunningham-Rundles, 2009; Warnatz et al., 2002). Despite the expansion of CD21^low^ B cells, autoimmune diseases were not prevalent in our patients. Moreover, antinuclear antibodies and rheumatoid factor were negative in all patients tested. While one of them (A.II.1) had oligoarticular arthritis as a teenager, her symptoms spontaneously resolved without immunomodulatory or specific treatment.

Based on all the above laboratory and clinical manifestations, flow cytometric screening by CD21 and CD23 expression on B cells (followed by genetic confirmation), PJP (± antibacterial) prophylaxis, IgG supplementation, hematologic malignancy monitoring, and consideration of hematopoietic stem cell transplantation as a possible curative option already proven efficient with other IKAROS-associated diseases (Kellner et al., 2019), summarize our experience-based recommendations for patients suffering from this form of AIOLOS-associated disease.

Identification of this AIOLOS*/IKZF3* mutation in patients with primary immunodeficiency/inborn errors of immunity (as previously shown with IKAROS mutations) highlights the critical role of IKAROS family members in lymphocyte development and the clinical consequences arising from genetic defects in their function. Similarities and differences between clinical and immunological phenotypes resulting from allelic variants in this family of transcription factors suggest that both AIOLOS and IKAROS act independently and also in concert when genetic variants alter their functions. Identification of this germline *IKZF3* mutation expands our understanding of the role of the IKAROS family of transcription factors in lymphocyte development, differentiation, and function and helps to establish a clinical phenotype of AIOLOS-associated diseases in humans.

## Materials and methods

### Patients and samples

All patients or their guardians provided written informed consent in accordance with the Declaration of Helsinki under institutional review board−approved protocols of the National Institute of Allergy and Infectious Diseases, National Institutes of Health. Blood from healthy donors was obtained under approved protocols.

### Next-generation sequencing and Sanger sequencing

WES was performed using genomic DNA extracted from peripheral blood mononuclear cells (PBMCs) from the index patient, her parents, paternal aunt, and paternal grandfather using the Illumina Exome Kit and HiSeq 2500 instrument according to the manufacturer’s protocols. Candidate variants were selected that were common to index patient, father, paternal aunt, and paternal grandfather but were not detected in the mother; novel or rare (nonsynonymous or indel, exonic, or splicing), in an immune-related gene with a gnomAD minor allele frequency ≤0.005 (0 homozygotes) and a combined annotation dependent depletion phred ≥20. The minor allele frequency, loss-of-function (LOF) observed/expected upper bound fraction, and probability of LOF were ascertained from the Genome Aggregation Database v.2.1.1 (gnomAD; Fadista et al., 2017; Kircher et al., 2014). The McDonald–Kreitman neutrality index was implemented in the Gene Damage Index server (Itan et al., 2015).

The WES-detected *IKZF3* mutation (NM_012481:c.479 A>G, p.N160S) was confirmed, and carrier testing of all available relatives was performed by Sanger sequencing (IKZF3_c.479_M13_F 5′-TGT​AAA​ACG​ACG​GCC​AGT​GAG​CTT​TTC​CCC​TAG​GCA​TC-3′; IKZF3_c.479_M13_R 5′-CAG​GAA​ACA​GCT​ATG​ACC​TTT​CTA​GTT​TCC​ACA​CTG​GGC-3′). The WES data have been deposited in NCBI’s Sequence Read Archive (accession no. PRJNA762368).

### Antibodies and reagents

The following antibodies were used in this study: anti-HA (BioLegend 901501 or Cell Signaling 3724), anti-Flag (Cell Signaling 14793 or 8146), anti-AIOLOS (Cell Signaling 15103), anti-Aiolos (9D10, EMD Millipore), anti-IKAROS (Cell Signaling 14859), anti-CD3 and anti-CD28 (eBioscience 16-0037-85 and 16-0289-85), β-actin (Cell Signaling 4970), anti-Gapdh (R&D Systems 2275-PC-100), normal rabbit IgG (Cell Signaling 2729), goat anti-rabbit IgG-HRP (Jackson Immunoresearch 111-035-003), goat anti-mouse IgG-HRP (Jackson Immunoresearch 115-035-003), donkey anti-rabbit IgG polyclonal IgG (GE Healthcare), anti-IgM (Jackson Immunoresearch 109-006-129), goat anti-rabbit IgG-Alexa Fluor 488 (Thermo Fisher Scientific A-11070), and goat anti-mouse IgG-Alexa Fluor 568 (Thermo Fisher Scientific A-11004). Protease and phosphatase inhibitor cocktail (PPC1010), PMA (P8139), and brefeldin A (B7651) were purchased from Sigma-Aldrich. Ionomycin (I24222) was purchased from Invitrogen. MEGACD40L (ALX522-110-C010) and ODN2006 (CpG, ALX-746-006-C100) were purchased from Enzo. IL-2 (200-02), IL-4 (200-04), and IL-21 (200-21) were purchased from PeproTech. Vectashield Mounting medium (H-1000) was purchased from Vector Laboratories.

Antibodies and clone names used for flow cytometry in human lymphocytes (purchased from either BD Bioscience or BioLegend except as noted) were CD3 (SK7), CD19 (HIB19), CD45RA (H100), CD45RO (UCHL1), CCR7 (G043H7), CD40L (TRAP1), CD27 (M-T271), CD38 (HB7), CD21 (B-ly4), kappa (TB2802), lambda (1–155-2), IgM (MHM-88), CD5 (L17F12), IFN-γ (B27), IL-4 (8D4-8), IL-17A (N49-653), CD40L (TRAP1), CD40 (5C3), CD69 (L78), IgD (polyclonal, Thermo Fisher Scientific H15501), and CD23 (Beckman Coulter 9P25). Antibodies and clone names used for flow cytometry for mouse lymphocytes were CD4 (RM4-5), CD8 (53-6.7), CD11b (M1/70), CD11c (HL3), CD19 (1D3), CD21 (7G6), CD23 (B3B4), CD38 (90), CD44 (IM7), CD62L (MEL-14), CD95 (Jo2), B220 (RA3-6B2), CXCR5 (2G8), IgM (R6-60), IgD (11-26c.2a), Ly6G (1A8), NK1.1 (PK136), PD-1(J43), Siglec-F (E50-2440), and TCRb (H57-597).

### Plasmid preparation

IKAROS (GenBank accession no. NM_006060) plasmids used in this study were previously described (Kuehn et al., 2016). Human AIOLOS (*IKZF3*) open reading frame clone (pcDNA3.1/C-(K)DYK-*IKZF3*; NCBI accession no. NM_012481) was purchased from GenScript and subcloned into the mammalian expression vector pcDNA3-HA. Indicated mutants for *IKZF3* were generated based on the site-directed mutagenesis protocol using AccuPrime Pfx DNA Polymerase, followed by DpnI treatment (Life Technologies).

### Immunoblotting

To generate T cell blasts, total PBMCs were stimulated with anti-CD3 and anti-CD28 in the presence of IL-2 (10 ng/ml, Peprotech) for 7 d. IL-2 was added every 2–3 d. T cell blasts were washed with PBS once and lysed in lysis buffer (50 mM Tris, pH 7.4, 150 mM NaCl, 2 mM EDTA, 0.5% Triton X-100, 0.5% NP-40, and protease and phosphatase inhibitor cocktail [Sigma-Aldrich]). Cell lysates were prepared and separated by NuPAGE Novex 4–12% Bis-Tris Protein Gels (Life Technologies) and transferred to nitrocellulose membranes using Trans-Blot Turbo Transfer system (Bio-Rad). The membranes were incubated with anti-AIOLOS and anti–β-actin. The images were acquired and analyzed with C-Digit scanner using Image Studio Software (Li-Cor).

For immunoblotting of mouse Aiolos, total thymocytes were isolated from mice and lysed with RIPA Lysis and Extraction Buffer (Thermo Fisher Scientific) supplemented with Halt Protease Inhibitor Cocktail (Thermo Fisher Scientific). Lysates were prepared and separated by 10% polyacrylamide gels (e-PAGEL, ATTO) and transferred to polyvinylidene difluoride membranes (EMD Millipore). After blocking with 5% (wt/vol) of skim-milk (Wako) in 1× TBS with Tween 20 buffer (Nacalai Tesque), the membrane was incubated with anti-Aiolos or Gapdh, followed by horseradish peroxidase­–conjugated second antibodies, and the target proteins were developed by use of Amersham ECL Select Western Blotting Detection Reagent and Amersham Imager 680 (GE Healthcare).

### IP

HEK293T cells were cotransfected with Flag-tagged AIOLOS (WT or N160S mutant) and HA-tagged AIOLOS or IKAROS WT using Effectene transfection reagent (Qiagen). 20–24 h after transfection, cell lysates were prepared in lysis buffer (50mM Tris, pH 7.4, 150mM NaCl, 2mM EDTA, 0.5% Triton X-100, and protease and phosphatase inhibitor cocktail [Sigma-Aldrich]). Total protein (500 µg) was incubated with rabbit anti-HA antibody or rabbit IgG (Cell Signaling). After 2-h incubation at 4°C on a rotating wheel, 50 µl Protein A/G-agarose beads (Pierce) were added and incubated for another hour. Beads were washed three times with lysis buffer, and the IP and total lysate samples were subjected to Western blotting with a mouse anti-HA antibody and mouse anti-FLAG antibody (Cell Signaling). Images were analyzed with Image Studio Software (Li-Cor).

### Cell culture

Either fresh Ficoll-isolated PBMCs or frozen PBMCs were cultured in RPMI 1640 with 10% FBS, 2 mM L-glutamine, 100 U/ml penicillin, and 100 µg/ml streptomycin (Thermo Fisher Scientific) at 37°C in a humidified 5% CO_2_ incubator. T cell blasts were generated as follows: PBMCs were stimulated with anti-CD3 and anti-CD28 (1 µg/ml each) in the presence of IL-2 (10 ng/ml; Peprotech) for 7–10 d. IL-2 was added every 2–3 d in RPMI 1640 with 10% FBS, L-glutamine, and penicillin/streptomycin. HEK293T (ATCC CRL-3216) and NIH3T3 (ATCC CRL-1658) cells were cultured in DMEM with 10% FBS, L-glutamine, and penicillin/streptomycin (Thermo Fisher Scientific).

### Fluorescence microscopy

NIH3T3 cells (0.8–1 × 10^5^) grown on coverslips in 6-well plates were transfected with indicated plasmids using Effectene (Qiagen) or Nucleofector kit R (Amaxa, program A-24) according to the manufacturer’s instructions. The cells were fixed, permeabilized, and stained with indicated antibodies, followed by fluorescence-conjugated secondary antibodies as previously described (Kuehn et al., 2021a). Cells were visualized using a ZOE fluorescent cell imager (Bio-Rad) or EVOS M5000 cell imaging system (Thermo Fisher Scientific). PC-HC foci in each transfected cell were counted using the image processing package Fiji (ImageJ) “find maxima” command (Schindelin et al., 2012).

### Lightshift chemiluminescent EMSA

HEK293T cells were transfected with Flag-AIOLOS WT or mutant alone or together with AIOLOS or IKAROS WT using Effectene (Qiagen) according to the manufacturer’s instructions. The nuclear extracts were prepared using NE-PER nuclear and cytoplasmic extraction kit (Thermo Fisher Scientific), and gel mobility shift assays were performed using LightShift Chemiluminescent EMSA kit (Thermo Fisher Scientific) according to the manufacturer’s instructions. IKBS1: forward, 5′-BIOTIN-TCAGCTTTTGGGAATACCCTGTCA-3′; reverse, 5′-BIOTIN-TGACAGGGTATTCCCAAAAGCTGA-3′).

### Evaluation of cell proliferation

Total PBMCs were incubated with CellTrace Violet cell proliferation kit according to the manufacturer’s instructions (1 µM; Invitrogen). Cells were stimulated with anti-CD3 antibody and anti-CD28 antibody (1 µg/ml each; eBioscience) for T cell proliferation. For B cell proliferation, cells were stimulated with anti-IgM (10 µg/ml; Jackson Immunoresearch), CD40L (100 ng/ml; Enzo), IL-4 (50 ng/ml; Peprotech), IL-21 (50 ng/ml; Peprotech), and CpG (500 nM; Enzo). 4 d after stimulation, cells were acquired and analyzed by flow cytometry (Becton Dickinson FACSCanto II) and FlowJo software (v10.7.2; TreeStar).

### Measurement of Th1, Th2, and Th17 populations

Total PBMCs were stimulated with PMA (100 ng/ml) and ionomycin (1 µM) for 5–6 h in the presence of brefeldin A (10 µg/ml). The stimulated cells were stained with CD3 and CD4 fluorochrome-conjugated antibodies for 30 min and then washed, fixed, and permeabilized with the BD Cytofix/Cytoperm kit. Cells were stained with the indicated antibodies (anti–IFN-γ [clone B27], anti–IL-4 [clone 8D4-8], and anti–IL-17A [clone N49-653]) and analyzed by flow cytometry.

### CD40L expression

CD4 T cells were enriched using EasySep human CD4 T cell enrichment kit (purity >90%; Stemcell Technology 19052). CD4 T cells were stimulated with PMA (100 ng/ml) and ionomycin (1 µM) for 20–24 h. Cells were stained with an anti-CD40L antibody (clone TRAP1) and analyzed by flow cytometry. CD69 (clone L78) was used as an activation marker.

### RNA-seq

RNA-seq was performed using RNA extracted from enriched T cell blasts (CD3 T cells were enriched from the T cell blasts, purity >90%; StemCell Technologies 19051) or naive B cells (purity >90%; Stemcell Technologies 19254) for the following study groups: healthy controls (*n* = 3–4) and patients with AIOLOS N160S (*n* = 1 for naive B cells [A.III.1] and *n* = 2 for T cell blasts [A.II.2 and A.III.1]). Two technical replicates were performed for each individual. Libraries were prepared using the AmpliSeq for Illumina Transcriptome Human Gene Expression panel, which captures 20,802 genes (>95% of human RefSeq genes). RNA-seq was performed on the Illumina HiSeq 2500 (Illumina; AmpliSeq for Illumina/HiSeq 2500). Demultiplexed reads were mapped to the hg19 genome using the splice-aware aligner Tophat (Trapnell et al., 2009). Gene-level count data were generated using the Rsubread feature counts, a read summarization program that counts mapped reads for genomic features such as genes (Liao et al., 2019). Differential expression analysis was performed using R (v.3.5.3) and DESeq2 (v.1.22.2; Love et al., 2014). We contrasted the AIOLOS study groups with the healthy donor group using the Wald test. The cutoff for significantly differentially expressed genes was >|−2| log2 fold expression and adjusted Wald test P < 0.01. Data were variance-stabilized for the Pearson correlation and heatmap analysis. Lists of differentially expressed genes for each study group vs. healthy controls were compared using Venny 2.0 (https://bioinfogp.cnb.csic.es/tools/venny/index.html), and immune-related genes were identified using GeneDistiller. Differentially expressed gene expression profiles for the patients with AIOLOS N160S mutations and a previous study (IKAROS N159S; Kuehn et al., 2021a) were analyzed using IPA analysis. Differentially expressed genes were analyzed using QIAGEN’s IPA (https://digitalinsights.qiagen.com/products-overview/discovery-insights-portfolio/analysis-and-visualization/qiagen-ipa/). Briefly, differentially expressed genes for each dataset and the corresponding log_2_ fold-changes and P values were uploaded into the IPA system for core analysis. Patient profiles were compared using IPA’s Diseases and Functions analyses. Fisher’s exact test was used to ascertain effected biology (diseases and biological functions) and the directional change of that effect (activation Z-score). Gene heatmaps with hierarchical clustering were generated to highlight similar expression patterns among group members (patients vs. controls). The RNA-seq data have been deposited in NCBI’s Gene Expression Omnibus (accession no. GSE183966)

### ChIP-seq

Enriched total CD3 T cells (CD3 T cell purity >90%; Stemcell Technologies 19051) from the index patient (A.III.1) and her father (A.II.2) and two healthy controls were subjected to ChIP-seq. Chromatin extraction, IP using AIOLOS antibody (Cell Signaling), library preparation, sequencing, and differential analysis based on peak signal fold-change were performed at Active Motif (Carlsbad, CA). MaxTags was used to detect differential occupation of regions within genes for T cells. The significance cutoff was log2 ratio > (+1) or < (−1), i.e., > twofold changes up or down. Genes with significant fold-changes were uploaded for Qiagen’s IPA (https://digitalinsights.qiagen.com/products-overview/discovery-insights-portfolio/analysis-and-visualization/qiagen-ipa/) to identify enriched biological functions. Patient profiles were compared using IPA’s Diseases and Functions analyses. Fisher’s exact test was used to ascertain affected biology (diseases and biological functions) and the directional change of that effect (activation Z-score). Gene heatmaps with hierarchical clustering were generated to highlight similar expression patterns among group members (patients vs. controls). ChIP peaks were identified using MACS2 (v2.1.1) using default parameters. The top 1,000 highly enriched peaks in each sample were subjected to binding motif analysis by HOMER (v4.10.3) with region size set to 50 bp. The genome-wide distribution of the peaks was analyzed using ChIPPeakAnno package (v3.26.2) in R. Binding affinity heatmap and correlation heatmap by AIOLOS in each sample were generated using Diffbind package (v2.2.12) in R.

### Generation of Ikzf3^N159S^ mouse strain

The *Ikzf3^N159S^* mutant allele was generated by CRISPR-Cas9–mediated genome editing. Crispr RNAs (crRNAs) that would target exon 4 of the *Ikzf3* gene and single-stranded donor oligonucleotides were synthesized at Integrated DNA Technologies. The crRNA, Cas9 mRNA, and donor DNA were coinjected into the cytoplasm of C57BL6/J fertilized eggs by the Animal Facility Group at RIKEN Center for Integrative Medical Sciences. Founder offspring that harbored the mutant allele were selected and crossed with WT C57BL6/J mice to establish the *Ikzf3*^*N159S*^ mouse line. Two representative lines of four F0 founders were confirmed to have the designed *Ikzf3^N159S^* mutation, and the mice were bred and analyzed with littermate controls. The sequences of crRNA and donor DNA are as follows: crRNA, 5′-GCAGTTTAATATGACGGAGAGG(PAM)-3′; donor DNA, 5′-CAC​TTA​CCT​TGT​GAT​CAG​CGA​TCT​CCT​TTT​TCC​TCC​TTT​CTG​AAG​GCG​AAC​GCC​CGT​TCC​AGT​GTA​ATC​AGT​GCG​GGG​CAT​CTT​TTA​CTC​AGA​AAG​GTA​GTC​TCC​TCC​GTC​ATA​TTA​AAC​TGC​ACA​CGG​GGG​AAA​AAC​CTT​TTA​AGT​GTC​ACC​TCT​GCA​ACT​ACG​CAT​GCC​AAA​GGA​GAG​ATG​CGC​TCA​CGG​GAC​ACC​TT-3′. All mice were maintained in the animal facility at RIKEN Center for Integrative Medical Sciences, and all animal procedures were in accordance with institutional guidelines for animal care and the protocol approved by the Institutional Animal Care and Use Committee of RIKEN Yokohama Branch (2020-026).

### Flow analysis for mouse study

Single-cell suspensions from thymus, spleen, and Peyer’s patch were prepared by mashing tissues through a 70-µm cell strainer (BD Bioscience). Single-cell suspensions were stained with appropriate antibodies and analyzed using a BD FACSCanto II (BD Bioscience), and data were analyzed using FlowJo software (BD Bioscience).

### Immunoglobulin measurement

Concentrations of mouse immunoglobulin were measured by mouse IgM (E99-101), IgG (E99-131), and IgA (E99-103) ELISA kits from Bethyl Laboratories.

### Statistical analysis

When indicated, data were analyzed using unpaired Student’s *t *test, Mann–Whitney *U* test, or Kruskal–Wallis test using Prism software (v6.0c; GraphPad). The differences were considered significant at P < 0.05.

### Online supplemental material

Fig. S1 shows CD40 expression on B cells and TCR-induced T cell proliferation. Fig. S2 shows RNA-seq analyses from T cell blasts. Fig. S3 shows ChIP-seq analyses from T cells. Fig. S4 shows the generation of *Ikzf*3*^N159S^* mouse strain and nonlymphoid immune cells in the mice. Fig. S5 shows the DNA binding and pericentromeric targeting of AIOLOS L162R. Table S1 lists candidate variants found in all four affected individuals. Table S2 shows fold-changes underlying the IPA Diseases and Functions analyses in Fig. S2, c and d, and Fig. S3 d.

## Supplementary Material

Table S1 lists candidate variants found in all four affected individuals.

Table S2 shows fold-changes underlying the IPA Diseases and Functions analyses in Fig. S2, c and d and Fig. S3 d.
