# Supplementary material for: T and B cell abnormalities, pneumocystis pneumonia, and chronic lymphocytic leukemia associated with an AIOLOS defect in patients
Source: J Exp Med. 2021 Oct 25;218(12):e20211118. doi: 10.1084/jem.20211118 (PMC8548914; doi:10.1084/jem.20211118)
Supplement: Table S1 — lists candidate variants found in all four affected individuals. [file JEM_20211118_TableS1.docx]

Table S1. Candidate variants in all four affected individuals

Variant selection criteria: Novel or rare variant (nonsynonymous or indel, exonic or splicing) in immune-related gene filtered with gnomAD MAF <=0.005 (0 homozygotes) and CADD phred >=20

Het: Heterozygous; ND: Not detected

gnomAD MAF: Genome Aggregation Database v.2.1.1 minor allele frequency for ALL populations

gnomAD LOEUF: loss-of-function [LOF] observed/expected upper bound fraction; Low LOEUF scores indicate strong selection against predicted loss-of-function (pLoF) variation in a given gene,

while high LOEUF scores suggest a relatively higher tolerance to inactivation

gnomAD pLI: Probability of loss-of-function (LoF) intolerance (pLI); LoF intolerant (pLI ≥0.9), LoF tolerant (pLI ≤0.1);

genes belonging to the haploinsufficient class are generally extremely LoF, thus pLI is the probability of belonging in the haploinsufficient class

LoFtool: The lower the LoFtool gene score percentile the more intolerant the gene is to functional variation (Fadista J et al. 2017)

Selective pressure: McDonald-Kreitman neutrality index implemented in the Gene Damage Index server (Itan et al., 2015)

CADD phred: Whole-genome Combined Annotation-Dependent Depletion [CADD] phred score (Kircher et al., 2014);

CADD_phred >=20 predict that a variant is amongst the 1% most deleterious of all possible substitutions

GERP++: Genomic Evolutionary Rate Profiling score >=2 indicates truly constrained sites

SiPhy: SIte-specific PHYlogenetic analysis; higher scores indicate higher conservation

SIFT: Predicted deleteriousness of a variant, D[eleterious], T[olerated]

PolyPhen2: Predicted deleteriousness of a variant, D probably_damaging, P possibly_damaging, B benign

pLOF: Probability of loss-of-function, 'High' indicates high-confidence pLoF variant; includes nonsense, splice acceptor, and splice donor variants caused by single nucleotide changes
